# Methylation profiling and evaluation of demethylating therapy in renal cell carcinoma

**DOI:** 10.1186/1868-7083-5-16

**Published:** 2013-09-13

**Authors:** Christopher J Ricketts, Mark R Morris, Dean Gentle, Salwati Shuib, Michael Brown, Noel Clarke, Wenbin Wei, Paul Nathan, Farida Latif, Eamonn R Maher

**Affiliations:** 1 Centre for Rare Diseases and Personalised Medicine, School of Clinical and Experimental Medicine, College of Medical and Dental Sciences, University of Birmingham, Birmingham B15 2TT, UK; 2 School of Applied Sciences University of Wolverhampton, Wolverhampton WV1 1SV, UK; 3 Department of Pathology, Universiti Kebangsaan Malaysia, Jalan Yaacob Latif, Bandar Tun Razak, 56000, Kuala Lumpur, Malaysia; 4 Institute for Cancer Sciences, Cancer Research UK Paterson Institute for Cancer Research, Manchester Academic Health Science Centre, University of Manchester, Manchester M20 4BX, UK; 5 The Christie Hospital, Wilmslow Road, Manchester M20 4BX, UK; 6 School of Cancer Sciences, University of Birmingham, Birmingham, UK; 7 Mount Vernon Cancer Centre - Medical Oncology, Rickmansworth Road, Northwood, Middlesex HA6 2RN, UK; 8 West Midlands Region Genetics Service, Birmingham Women’s Hospital, Edgbaston, Birmingham B15 2TG, UK; 9 Department of Medical Genetics, University of Cambridge, Addenbrooke’s Treatment Centre, Cambridge Biomedical Research Campus, Cambridge CB2 0QQ, UK

**Keywords:** Renal cancer, Epigenetics, Methylation, Demethylation, VHL, Renal cell carcinoma, Methylation, Therapy

## Abstract

**Background:**

Despite therapeutic advances in targeted therapy, metastatic renal cell carcinoma (RCC) remains incurable for the vast majority of patients. Key molecular events in the pathogenesis of RCC include inactivation of the *VHL* tumour suppressor gene (TSG), inactivation of chromosome 3p TSGs implicated in chromatin modification and remodelling and *de novo* tumour-specific promoter methylation of renal TSGs. In the light of these observations it can be proposed that, as in some haematological malignancies, demethylating agents such as azacitidine might be beneficial for the treatment of advanced RCC.

**Results:**

Here we report that the treatment of RCC cell lines with azacitidine suppressed cell proliferation in all 15 lines tested. A marked response to azacitidine therapy (>50% reduction in colony formation assay) was detected in the three cell lines with *VHL* promoter methylation but some RCC cell lines without *VHL* TSG methylation also demonstrated a similar response suggesting that multiple methylated TSGs might determine the response to demethylating therapies. To identify novel candidate methylated TSGs implicated in RCC we undertook a combined analysis of copy number and CpG methylation array data. Candidate novel epigenetically inactivated TSGs were further prioritised by expression analysis of RCC cell lines pre and post-azacitidine therapy and comparative expression analysis of tumour/normal pairs. Thus, with subsequent investigation two candidate genes were found to be methylated in more than 25% of our series and in the TCGA methylation dataset for 199 RCC samples: *RGS7* (25.6% and 35.2% of tumours respectively) and *NEFM* in (25.6% and 30.2%). In addition three candidate genes were methylated in >10% of both datasets (*TMEM74* (15.4% and 14.6%), *GCM2* (41.0% and 14.6%) and *AEBP1* (30.8% and 13.1%)). Methylation of *GCM2* (*P* = 0.0324), *NEFM* (P = 0.0024) and *RGS7* (*P* = 0.0067) was associated with prognosis.

**Conclusions:**

These findings provide preclinical evidence that treatment with demethylating agents such as azacitidine might be useful for the treatment of advanced RCC and further insights into the role of epigenetic changes in the pathogenesis of RCC.

## Background

Aberrant DNA methylation, in particular promoter hypermethylation and transcriptional silencing of tumour suppressor genes (TSGs), has an important role in the development of many human cancers including renal cell carcinoma (RCC), which accounts for approximately 2% of all cancers and is diagnosed in >200,000 individuals in the world each year [1-3]. Most RCC cases (approximately 75%) are classified as clear-cell (conventional) RCC (cRCC), and the most common genetic event in the evolution of sporadic cRCC is inactivation (by mutation, allele loss or promoter methylation) of the von Hippel-Lindau (*VHL*) TSG [4-7]. *VHL* inactivation leads to stabilization of the hypoxia-inducible transcription factors (HIF)-1 and HIF-2 and activation of a wide repertoire of hypoxia response genes [8,9]. Although the *VHL* mutations in primary clear-cell RCC were first described 20 years ago, until recently, attempts to identify other frequently mutated RCC genes had generally been disappointing. However the application of large candidate gene re-sequencing and exome re-sequencing strategies to sporadic RCC has led to the identification of several frequently mutated TSGs. Thus, mutations in *PBRM1*, which encodes a chromatin remodeling complex subunit, were detected in approximately 40% of RCC [10] and mutations in genes for other chromatin modifiers have also been described, such as *SETD2, KDM6A/UTX* and *BAP1*[11-13]. In addition, we and others have sought to investigate the role of *de novo* TSG promoter hypermethylation in the pathogenesis of RCC and evidence for tumour-specific promoter region hypermethylation has been reported for >60 candidate TSGs (see [2] and references within). Notably, certain important TSGs (for example, *RASSF1A*) are frequently methylated but infrequently mutated [14,15].

There are no curative therapies for metastatic RCC and an important rationale for cancer genomic and epigenomic studies is to provide a basis for the development of novel therapies for advanced disease. The identification of frequently mutated and methylated RCC TSGs could highlight critical pathways that might be targeted for therapeutic intervention but, if *de novo* promoter methylation of candidate RCC TSGs plays a significant part in renal oncogenesis, it can be proposed that reversal of promoter methylation would reduce RCC cell proliferation. Demethylating agents such as azacitidine (5-aza-cytidine/Vidaza) and decitabine (5-aza-2’-deoxycytidine/Dacogen) have been used with some success to treat myelodysplastic syndrome and acute myelogenous leukaemia [16] and are being investigated for the treatment of solid tumours (for example, of the breast and colon) [17].

Given the key role of the *VHL* TSG inactivation in RCC we investigated whether the response of RCC cell lines to treatment with the demethylating agent azacitidine was dependent on *VHL* methylation status. We found that azacitidine treatment suppressed the growth of RCC cell lines but the response to azacitidine was not restricted to *VHL* methylated cell lines, and so to identify novel epigenetically inactivated candidate TSGs for RCC we proceeded to analyse the results of combined copy number and methylation profiling on primary RCC tumours, and investigate whether methylation of such genes was associated with survival.

## Results

### *In vitro* dose-response relationships for azacitidine treatment and promoter region hypermethylation

To determine the effects of treatment with escalating doses of azacitidine (control, 0.3 μM, 1 μM, 3 μM) on *in vitro* cell growth, colony formation assays were undertaken with fifteen RCC cell lines (three with VHL promoter region methylation (SKRC54, 769-P and A704)). After initial optimisation experiments, cells were seeded at 1 in 2,000 dilution and maintained in DMEM and 10% fetal bovine serum; surviving colonies were counted 14 to 21 days after initial seeding.

**Figure 1:**
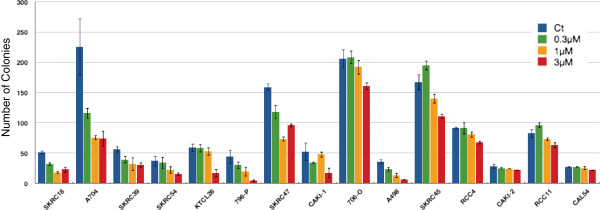
**Azacitidine treatment and growth of renal cell carcinoma (RCC) cell lines.** Effects of escalating doses of azacitidine on colony formation (absolute numbers of colonies) for 15 RCC cell lines. Treatment with 3 μM azacitidine reduced the colony-forming potential of all RCC cell lines. However, at lower doses the effect varies markedly between cell lines.

The results of the colony formation assays are displayed in Figure 1. Compared to controls, treatment with 3 μM azacitidine reduced the number of colonies formed in each cell line to varying degrees ranging from >90% reduction in 769-P to <20% reduction in CAL54. However the sensitivity to lower doses of azacitidine was variable with some cell lines demonstrating no effects at the lowest 0.3 μM azacitidine doses (for example, 786-O, SKRC45, RCC4 and RCC11), while four cell lines, SKRC18, A704, 796-P and A498, demonstrated a 50% or greater reduction at both 1 μM and 3 μM azacitidine.

### Effect of azacitidine on anchorage-independent growth of RCC cell lines

**Figure 2:**
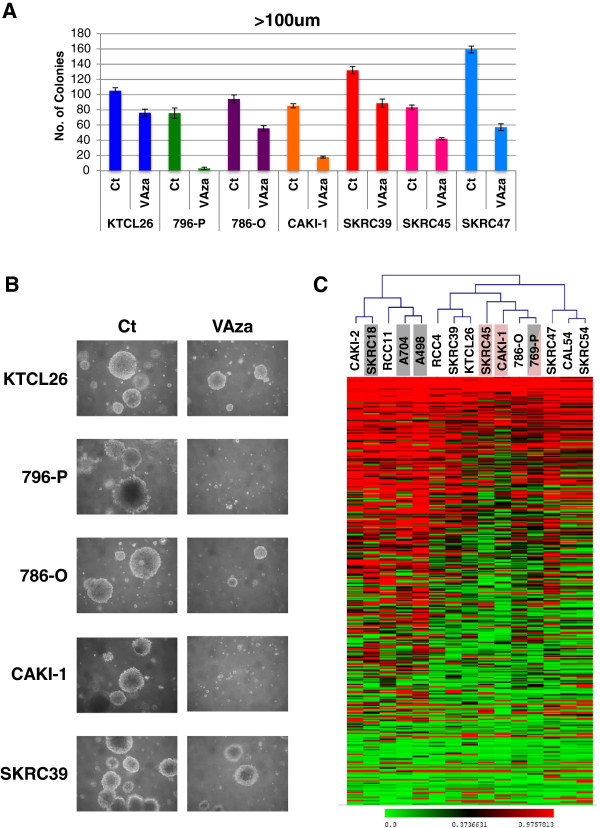
**Azacitidine treatment reduces the anchorage independent growth of renal cell carcinoma (RCC) cell lines. (A)** Pre-treatment with 3 μM azacitidine resulted in reduction of anchorage-independent growth in all seven RCC cell lines tested. Other cell lines did not grow suspended in agar. Error bars represent standard error. **(B)** Representative images (6 weeks after seeding in agar) of control colonies and colonies formed after azacitidine pre-treatment. **(C)** Illumina GoldenGate Methylation Cancer Panel I Beadarray cluster analysis results of RCC cell lines. The four cell lines, SKRC18, A704, 796-P and A498 that demonstrated a 50% or greater colony reduction at both 1 μM and 3 μM azacitidine doses are highlighted with grey boxes. The three cell lines with the greatest (>50%) reduction in anchorage-independent colony formation, SKRC45, CAKI-1 and 769-P, are highlighted with red boxes.

The effect of 3 μM azacitidine on anchorage-independent growth of seven RCC cell lines was assessed in soft agar assays (the remaining cell lines did not grow in soft agar). In all cases treatment with 3 μM azacitidine reduced anchorage-independent growth (as assessed by number of colonies >100 μm) (Figure 2A and B). The median reduction (relative to control) in number of colonies was 49.7% (range 27.6 to 96.9%). The only cell line from the four most azacitidine-sensitive lines that could be assessed, 769-P also demonstrated the greatest degree of reduction.

### Relationship between CpG methylation profiling of human cancer genes and RCC cell line growth response to azacitidine treatment

The Illumina GoldenGate Methylation Cancer Panel I array provides quantitative CpG methylation data at 1,505 individual CpG dinucleotides associated with 807 human genes (with enrichment for candidate TSGs methylated in human cancers). In order to profile patterns of RCC-specific candidate TSG methylation after excluding genes methylated in normal kidney tissue (including X-chromosome genes), cluster analysis was performed to group the RCC cell lines according to methylation profiling results (see Figure 2C). This produced two major clusters. The first cluster contained five cell lines and included three of the four of the most azacitidine-sensitive lines (SKRC18, A704 and A498, but not 769-P (Figure 2C, grey boxes)), though the other two lines in this cluster, CAKI-2 and RCC11, demonstrated much milder responses to azacitidine. The three cell lines with the greatest (>50%) reduction in anchorage-independent colony formation, SKRC45, CAKI-1 and 769-P were in the second major cluster and apparently did not demonstrate a strong hypermethylation phenotype (Figure 2C, red boxes).

### Copy number and CpG methylation array analysis of sporadic primary RCCs

**Figure 3:**
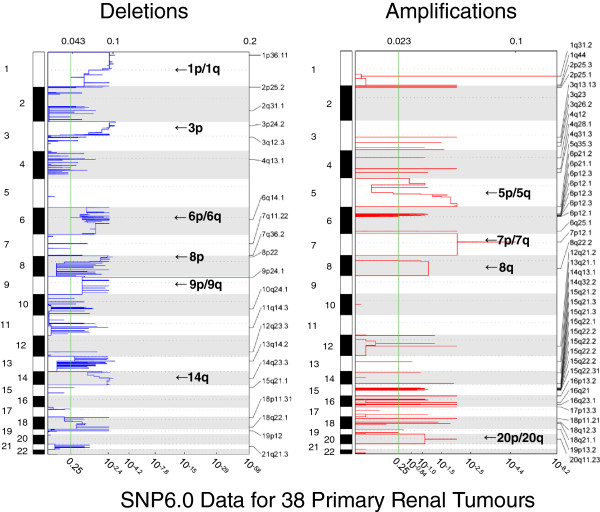
**Copy number analysis.** Genetic identification of significant targets in cancer (GISTIC) analysis of the Affymetrix SNP6.0 data for 46 sporadic renal cell carcinoma (RCC) tumours, highlighting the large regions of chromosomes that were either deleted or amplified/duplicated to a significant degree. Smaller specific regions of loss or gain are highlighted to the right of each analysis

Copy number analysis of 46 primary sporadic RCCs demonstrated multiple regions of chromosomal abnormality in the tumour genomes. Large-scale deletions of statistical significance were observed at 1p/1q, 3p, 6p/6q, 8p, 9p/9q and 14q, and large-scale amplifications/duplications of statistical significance were observed at 5p/5q, 7p/7q, 8q and 20p/20q (Figure 3). These results were largely in agreement with previous similar studies that had highlighted chromosomal deletions at 3p, 14p, 8p, 6q, 9p and 4p [18], and losses in these regions occurred at 74, 36, 24, 20, 19 and 13% of tumours respectively, whereas previously published chromosomal amplifications/duplications include 5q and 7 and these occurred in 43 and 26% of cases respectively (Additional file 1: Table S1).

**Figure 4:**
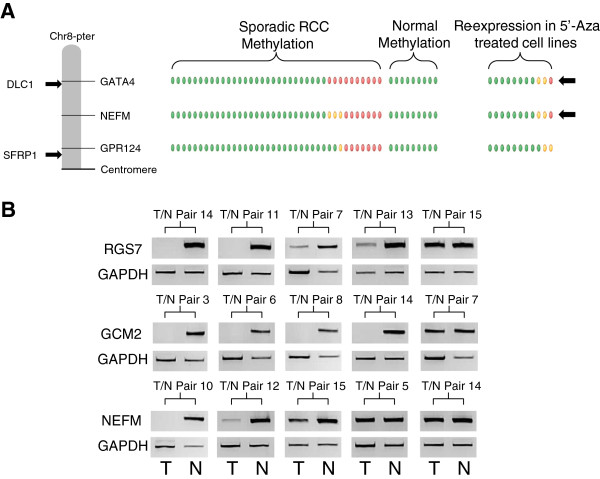
**Analysis of the deleted chromosomes. (A)** Schematic of the deleted chromosome 8p. The methylation levels for the selected genes in the 38 sporadic renal cell carcinomas (RCCs) are represented by green ovals for no significant methylation or yellow (β-values ≥0.33) and red (β-values ≥0.4) ovals for positive levels of hypermethylation. The methylation levels for the nine normal kidneys are represented by green ovals for no methylation (β-values <0.25). The degree of re-expression after 5′-aza treatment in the 11 RCC cell lines is represented by green ovals for no significant increase/change or yellow ovals for a positive increase (4-fold or greater) and red ovals for a highly positive increase (8-fold or greater). Other genes of interest were mapped to the chromosome with black arrows representing known hypermethylated RCC-associated genes and blue arrows representing genes known to be mutated in RCC. **(B)** Example reverse-transcription (RT)-PCR analysis of the tumour/associated kidney normal pairs for the positive selected genes from chromosomes 1, 6 and 8p. Each pair for each gene is shown with the tumour result on the left and the associated normal on the right and with a glyceraldehyde-3-phosphate dehydrogenase (GAPDH) band to demonstrate loading. Generally, the loading was skewed so that more tumour cDNA was analysed than the associated normal cDNA to emphasise that any loss observed was real and significant. In all cases at least one pair was shown where no loss/decrease was observed, to demonstrate the mRNA would normally be expressed in the tumour tissue.

**Figure 5:**
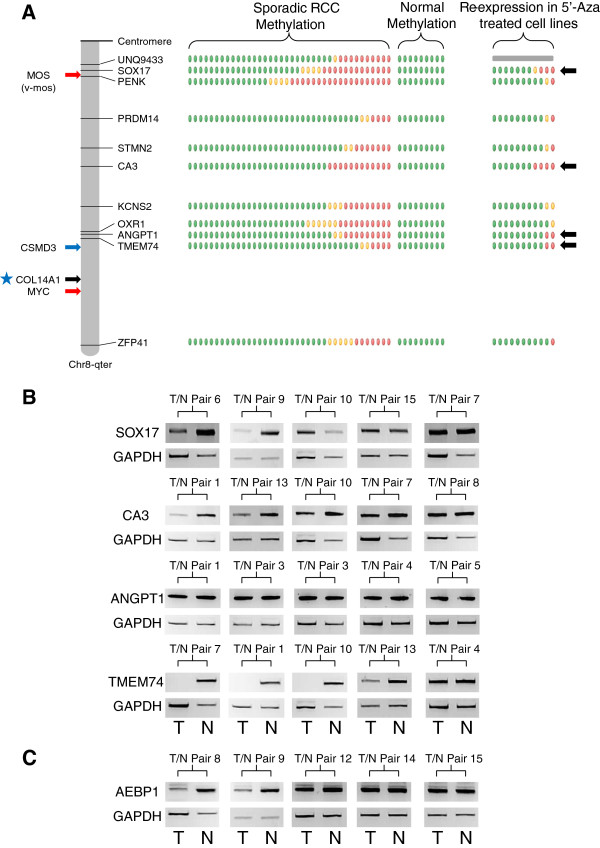
**Analysis of the amplified/duplicated chromosomes. (A)** Schematic of the amplified/duplicated chromosome 8q. The methylation levels for the selected genes in the 38 sporadic renal cell carcinomas (RCCs) are represented by green ovals for no significant methylation or yellow (β-values ≥0.33) and red (β-values ≥0.4) ovals for positive levels of hypermethylation. The methylation levels for the nine normal kidneys are represented by green ovals for no methylation (β-values <0.25). The degree of re-expression after 5′-aza treatment in the 11 RCC cell lines is represented by green ovals for no significant increase/change or yellow ovals for a positive increase (4-fold or greater) and red ovals for a highly positive increase (8-fold or greater). Other genes of interest were mapped to the chromosome, with red arrows representing oncogenes, black arrows representing known hypermethylated RCC-associated genes and blue arrows representing genes known to be mutated in RCC. The blue star indicates the gene is both mutated and hypermethylated. **(B)** Example reverse transcription (RT)-PCR analysis of the tumour/associated kidney normal pairs for all the selected genes for chromosome 8q and **(C)** the positive gene on chromosome 7. Each pair for each gene is shown with the tumour result on the left and the associated normal on the right and with a glyceraldehyde-3-phosphate dehydrogenase (GAPDH) band to demonstrate loading. Generally, the loading was skewed so that more tumour cDNA was analysed than the associated normal cDNA to emphasise that any loss observed was real and significant. In all cases at least one pair was shown where no loss/decrease was observed to demonstrate the mRNA would normally be expressed in the tumour tissue.

In any given tumour a gene could be inactivated or lost by either copy number alterations, promoter methylation, somatic mutation or a combination of these factors and others. It is reasonable to hypothesize that genes that reside within areas of copy number variation and are frequently methylated may represent genes with important roles in tumourigenesis. In this study these were considered two independent mechanisms and were not assessed in combination. Thus, to identify genes demonstrating frequent tumour-specific promoter region methylation (using criteria of six or more tumours with β-values ≥0.33 and β-values <0.25 in all nine normal samples) that mapped within the previously described statistically significant regions of copy number abnormalities, we reanalysed the results of tumour CpG methylation profiling using the Illumina Infinium HumanMethylation27 array [19]. There were 126 genes that satisfied the selection criteria: 72 mapped within the common chromosome deletion regions (1p/1q (n = 24), 3p (n = 5), 6p/6q (n = 23), 8p (n = 4), 9p/9q (n = 5) and 14q (n = 11) and 54 to the common amplified/duplicated chromosome regions (5p/5q (n = 18), 7p/7q (n = 16), 8q (n = 11) and 20p/20q (n = 11) (Figures 4A and 5A, and Additional file 2: Figures S1A-D and Additional file 3: Figure S2A-B). Four genes (*DLEC1*, *COL1A2*, *FBN2* and *TNFRSF10C*) that satisfied the selection criteria were investigated in our previous study [19] and thus removed from this study.

To prioritise the 122 candidate novel methylated genes for further investigation we interrogated our previously reported data on gene expression post 5-aza-cytidine treatment in RCC cell lines [2] and identified those candidate methylated genes whose expression was upregulated after 5-aza-cytidine treatment, at least 8-fold in two cell lines or ≥8-fold in one cell line and ≥4-fold in at least another two cell lines. This refined the candidate gene list down to 37 genes: 19 genes on the deleted chromosomes (1p/1q (n = 9), 6p/6q (n = 3), 8p (n = 2), 9p/9q (n = 1) and 14q (n = 4)) and 18 genes on the amplified/duplicated chromosomes (5p/5q (n = 7), 7p/7q (n = 4), 8q (n = 4) and 20p/20q (n = 3)) (Figures 4A and 5A, and Additional file 2: Figure S1A-D and Additional file 3: Figure S2A-B). To determine whether tumour-specific promoter methylation was associated with suppression of gene expression in primary tumours, RT-PCR was performed on 15 tumours and their associated normal pairs for the 37 genes selected for further investigation. Five genes demonstrated complete loss or downregulation of expression in at least 20% of RCC tumours (*GCM2* (80% of RCC), *RGS7* (46.7%), *TMEM74* (46.7%), *NEFM* (20%) and *AEBP1* (20%)) (Table 1, Figures 4B and 5B-C). *GCM2*, *RGS7* and *NEFM* were located in regions of deletion and *TMEM74* and *AEBP1* in copy number gain regions (Table 1, Figures 4A and 5A, and Additional file 2: Figure S1A,C and Additional file 3: Figure S2B).

**Table 1:** RT-PCR analysis for selected genes in chromosomal regions of deletion or amplification/duplication in primary RCC tumours and associated normal tissue

**Deletions**					
**Chromosome**	**Potential tumour specifically inactivated genes**				
	Tum Exp. Lost/Tum. Exp. Down-regulated/Tum. Exp. Normal or Up-regulated (Percentage of lost/down-regulated)				
Chromosome 1	CHD5	TNFRSF1B	KIF17	UQCRH	GPX7
	0/1/14 (6.7%)	0/0/15 (0.0%)	0/0/15 (0.0%)	0/0/15 (0.0%)	0/0/15 (0.0%)
	TTC22	PDE4DIP	**RGS7**	TRIM58	
	1/1/13 (13.3%)	0/0/15 (0.0%)	3/4/8 (46.7%)	0/2/13 (13.1%)	
Chromosome 6	**GCM2**	HIST1H3G	HIST1H4H		
	8/0/2 (80.0%)	0/0/15 (0.0%)	0/0/15 (0.0%)		
Chromosome 8p	GATA4	**NEFM**			
	1/0/8 (11.1%)	1/2/12 (20.0%)			
Chromosome 14q	EFS	PTGDR	C14orf39	FLRT2	
	0/1/14 (6.7%)	0/1/14 (6.7%)	1/0/8 (11.1%)	0/1/14 (6.7%)	
**Amplifications**					
**Chromosome**	**Potential tumour specifically inactivated genes**				
	(Percentage of tumours in which gene expression is lost or down-regulated in brackets)				
Chromosome 1	TWIST1	HOXA11	AEBP1	KLF14	
	1/0/13 (7.1%)^1^	0/2/13 (13.3%)	3/0/12 (20.0%)	0/2/13 (13.3%)	
Chromosome 8p	SOX17	CA3	ANGPT1	TMEM74	
	0/2/13 (13.3%)	0/3/12 (20.0%)	0/0/15 (0.0%)	4/3/8 (46.7%)	

^1^Key= Tumour Expression Lost/Tumour Expression Down-regulated/Tumour Expression Normal or Up-regulated (Percentage of lost/down-regulated).

Genes in bold type (*GCM2*, *RGS7* and *NEFM*) demonstrated complete loss or downregulation of expression in at least 20% of RCC tumours and were located in regions of deletion.

**Figure 6:**
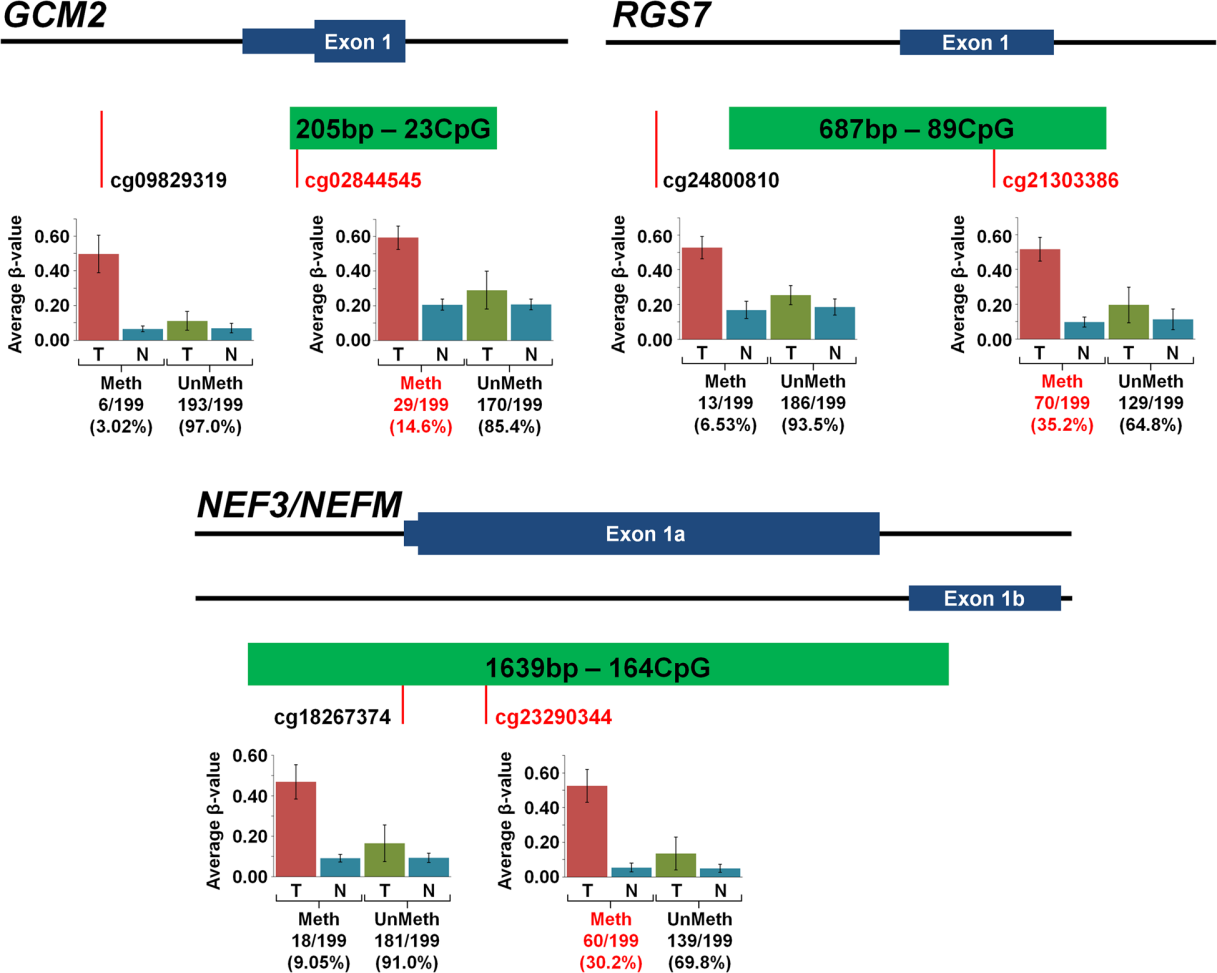
**Assessment of selected methylated Infinium Methylation27 probes in the Cancer Genome Atlas (TCGA) tumour and associated normal pairs.** These in-scale diagrams map the position of the Infinium Methylation27 microarray probes for three of the five selected genes, *GCM2*, *RGS7*, *NEFM*, in relation to their CpG island and first exon(s). The probes selected for by this analysis are coloured red. For each probe there is a graph of the average β-values for both the tumour and the associated normal with the 199 tumour/associated normal TCGA samples split into those designated methylated or unmethylated. Methylated samples were defined as having an increase in β-value of 0.3 or greater within the tumour compared to the associated normal. The number and percentage of methylated and unmethylated samples are shown, with those demonstrating significant tumour-specific methylation coloured in red.

**Figure 7:**
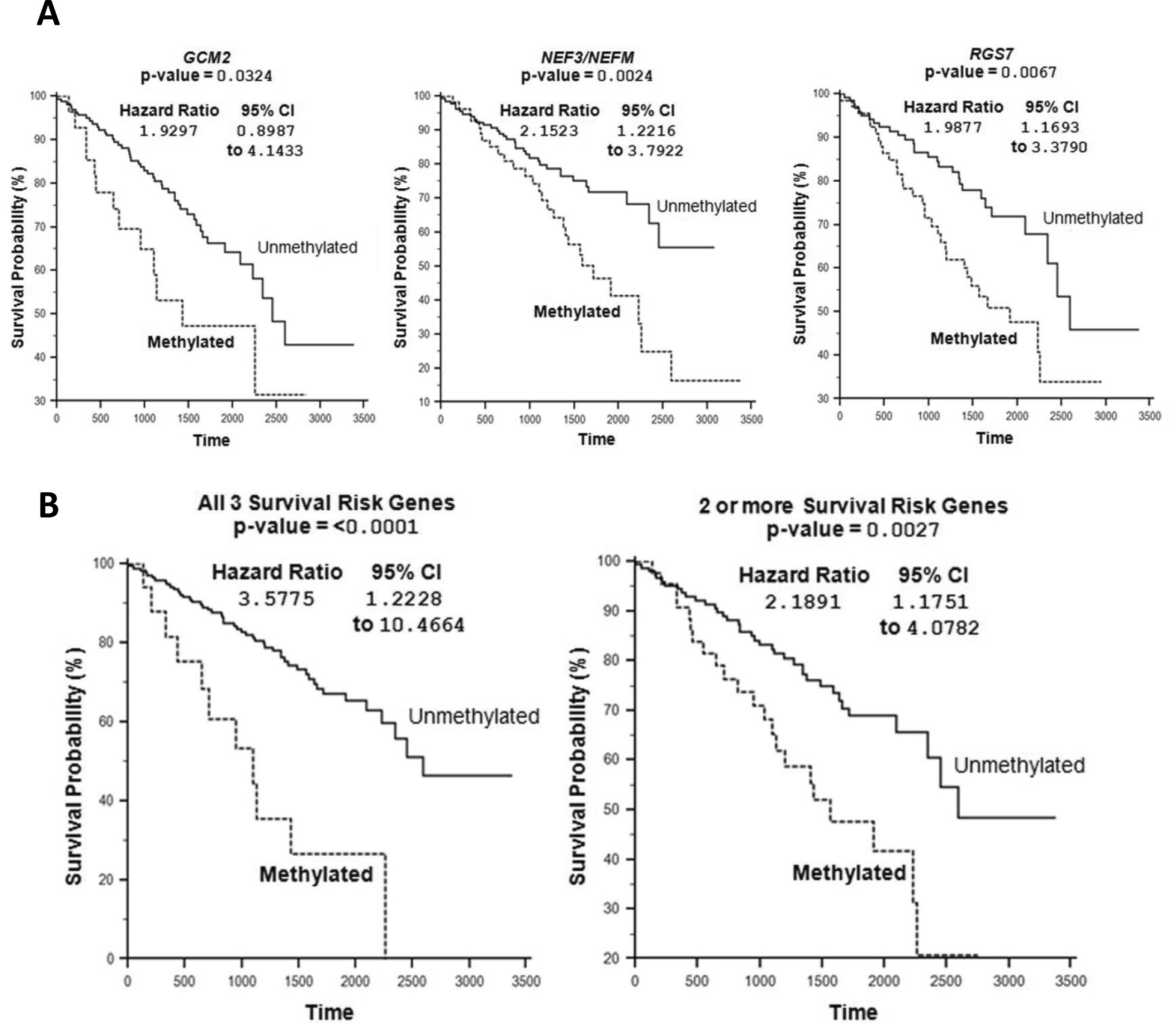
**Kaplan-Meier survival curves for the Cancer Genome Atlas (TCGA) samples dependent on the methylation of the selected gene probes. (A)** These Kaplan-Meier survival curves demonstrate the difference in survival between methylated and unmethylated tumours for individual gene probes for *GCM2*, *NEFM* and *RGS7*. **(B)** These Kaplan-Meier survival curves demonstrate the difference in survival between methylated and unmethylated tumours for methylation of all three or two or more of these genes **(B)**.

To replicate these findings we investigated whether the five candidate methylated genes (*GCM2*, *RGS7*, *TMEM74*, *NEFM* and *AEBP1*) were also methylated in the 199 tumour and normal pairs analysed on the same platform (Illumina HumanMethylation27 array) by the Cancer Genome Atlas (TCGA) Kidney renal clear cell carcinoma (KIRC) project. A difference in β-value of 0.3 or greater between tumour and normal tissue was taken to represent a significant increase in methylation. In the TCGA data the frequency of methylation for *RGS7* was 35.2% (Birmingham series methylation frequency = 25.6%), for *NEFM* it was 30.2% (versus 25.6%), for *TMEM74* it was 14.6% (15.4%), for *GCM2* it was 14.6% (versus 41%) and for *AEBP1* it was 13.1% (30.8%) (Figure 6 and Additional file 4: Figure S3). Clinical data were available for most (193/199) of the TCGA samples and we therefore investigated whether the methylation status of *GCM2*, *RGS7*, *TMEM74*, *NEFM* and *AEBP1* correlated with clinical characteristics (Additional file 5: Table S2). Methylation at *GCM2* (*P* = 0.0324), *NEF3/NEFM* (*P* = 0.0024) or *RGS7* (P = 0.0067) was significantly associated with survival (Figure 7A and Additional file 6: Figure S4). The combined effect of the presence of methylation in all genes demonstrated a better association with survival (*P* <0.0001) and the combination of any two also being significantly associated (*P* = 0.0027) (Figure 7B).

## Discussion

Demethylating agents such as azacitidine or decitabine have been used successfully for the treatment of haematological neoplasias, such as acute myeloid leukaemia, chronic myelomonocytic leukaemia (CMML) and the preleukaemic disorder myelodysplastic syndrome [16]. Additionally, usage of these demethylating agents is being extended to solid tumours in clinical trials and the National Institutes of Health (NIH) currently have active trials for azacitidine in non-small cell lung cancer and squamous cell head and neck cancer, and for decitabine in ovarian cancer and melanoma (http://clinicaltrials.gov). Currently, epigenetic abnormalities are being recognised as a prominent feature of RCC. Thus, mutations in genes implicated in chromatin remodelling and modification such as *PBRM1, SETD1, KDM6A/UTX* and *BAP1* have recently been shown to be frequently found in RCC [10-13] and more than 60 candidate TSGs have been reported to demonstrate promotor region methylation in RCC [2] (and references within). In our *in vitro* studies of RCC cell lines we found that treatment with azacitidine reduced cell growth, in both colony formation assays and soft agar assays, in all cell lines tested. These findings are consistent with two previous studies in RCC cell lines. Thus Alleman *et al*. [20] treated four RCC cell lines (with promoter methylation and loss of expression of *VHL*) with 5-aza-2′-deoxycytidine and demonstrated re-expression of VHL and reduced size of xenografted tumours. In addition, Negrotto *et al*. [21] reported that non-cytotoxic doses of decitabine administered to four RCC cell lines (SKRC29, SKRC45, ACHN and RENCA) decreased proliferation of RCC cells (in *in vitro* and *in vivo* assays) but not normal kidney epithelial cells. In our studies the reduction in cell growth was variable with some cell lines showing reduction at lower doses and marked variations in relative response (compared to untreated growth) to 1 μM and 3 μM azacitidine. Each of the cell lines with *VHL* TSG promoter methylation demonstrated a marked (>50%) reduction in growth in the colony formation but an association between VHL methylation and response to azacitidine was not statistically significant (*P* = 0.077). Given the critical role of *VHL* inactivation in RCC, a marked response to demethylating therapy in cell lines with *VHL* hypermethylation might not appear unexpected, and indeed, preferential effects of 5-aza-2′-deoxycytidine treatment of *VHL*-methylated versus *VHL*-mutated but unmethylated cell lines were described in *in vivo* studies [20]. However, previously re-expression of pVHL in *VHL*-inactivated RCC cells has been reported to suppress growth in *in vivo* but not *in vitro* assays [22,23]. It is possible that the better response in *VHL*-methylated cell lines might be because *VHL*-methyation is a marker for other critical epigenetic changes that are reversed by treatment with azacitidine. However, there were no apparent clear correlations between response to azacitidine and the grouping of RCC cell lines according to their methylation array profiles (although this only represents a subset of CpGs) or cell-line population doubling-time.

In order to expand the catalogue of frequently methylated candidate TSGs in RCC we undertook an analysis of copy number and CpG methylation profiling of sporadic RCC. Previously we reported an analysis of the CpG methylation analysis data [19] but for this reanalysis we concentrated on the methylation status of genes within regions that demonstrated copy number abnormalities. One caveat to this methodology is the interpretation of deletions or amplifications at centromeric and telomeric regions where enrichment for variable non-tandem repeats have been shown to harbour hotspots of natural variation in the normal population [24-26]. For this study we only considered large, common deletions or amplifications that had been previously observed rather than smaller focal alterations to avoid this. When hunting for novel TSGs, chromosomal regions that demonstrate copy number loss in cancer have traditionally been prioritised, whereas regions of copy number gain are highlighted in the search for proto-oncogenes. However, we decided to focus on both regions of copy number gain and loss, as we reasoned that if a region of copy number gain provided a growth advantage for a cancer cell there might need to be concurrent inactivation of TSGs within that region of amplification to gain the full benefit. Indeed, two of the five novel candidate TSGs (*TMEM74* and *AEBP1*) that we focused on mapped within regions of copy number gain. These five genes represent a diversity of functions. *AEBP1* (the adipocyte enhancer binding protein 1) is a transcriptional repressor with carboxypeptidase (CP) activity. AEBP1 may regulate mitogen-activated protein (MAP)-kinase activity and has been reported to be methylated in rat prostate cancer lines [27]. *GCM2* is homologous to the Drosophila glial cells missing gene and encodes a transcription factor implicated in parathyroid gland development, and mutations in *GCM2* have been reported in familial hypoparathyroidism [28,29]. *NEFM* encodes the medium neurofilament protein and has been reported previously to be methylated in pancreatic cancers and astrocytoma [30,31]. The regulator of G-protein signalling (RGS) pathway has been reported to play a role in signalling transduction and carcinogenesis and a *RGS7* single nucleotide polymorphism (SNP) has previously been reported to be associated with survival in non-small cell lung cancer patients treated with chemoradiotherapy [32]. TMEM74 is known to regulate autophagy and though it has not been implicated previously in RCC, it is interesting to note that the tumour suppressor activity of the *VHL* TSG has been linked to regulation of autophagy [33-35].

A feature of the present study is that we were able to corroborate our CpG methylation profiling results with those generated by the TCGA on the same platform. However, though the direct assay of CpG methylation facilitates the identification of possible epigenetically inactivated TSGs, we have demonstrated previously that this strategy is not entirely specific as some genes with apparent tumour-specific methylation are not confirmed on further analysis [19]. Hence, it is necessary to combine, as in the current study, high-throughput methylation profiling assays with confirmatory assays and investigations to confirm transcriptional silencing. Having pursued such investigations we were able, in the knowledge that the probes used to detect methylation were accurate, utilise the TCGA data to look for correlations between gene-specific methylation and survival. We found significant correlations between survival and methylation at *GCM2*, *NEFM* and *RGS7*. *De novo* tumour-specific promoter methylation represents an attractive target for developing biomarkers, as methylated tumour DNA can be detected in plasma and urine [36,37] and the methodology to detect CpG promoter TSG methylation is more straightforward than that required to detect the wide variety of inactivating mutations that usually occur in TSGs. Hence, the identification of methylated candidate TSGs that are associated with prognosis will facilitate the development of a repertoire of biomarkers that might be used to stratify RCC into tumours with good or poor prognosis.

## Conclusions

Previously we and others have reported evidence that the frequency of TSG methylation is not equally distributed across RCC and that a subset of tumours may manifest high levels of TSG methylation [19,38,39]. We did not find a correlation between the methylation profile of RCC cells lines and response to epigenetic therapy but it could be that more comprehensive methylation profiling is required to uncover such an effect. Alternatively it could be that therapeutic response to demethylating agents is determined by the methylation status of a number of key genes. Given the large number of candidate TSGs reported to be methylated in RCC and the relatively small number of available RCC cell lines it is clear that it will be difficult to reliably identify key genes whose methylation status could predict response to epigenetic therapies. Our results suggest that epigenetic therapies might provide an additional treatment option for metastatic RCC that is unresponsive to standard management options. In order to develop predictors of likely response to epigenetic therapies in RCC it is important that whenever possible, data on the methylation status of renal TSGs (for example, *VHL*) are collected on patients with advanced RCC who receive such therapy.

## Methods

### RCC cell lines, tumours and azacitidine treatment

Fifteen RCC cell lines (SKRC18, SKRC39, SKRC45, SKRC47, SKRC54, RCC4, RCC11, 786-O, 796-P, A704, A498, KTCL26, CAL54, CAKI-1 and CAKI-2) were maintained in DMEM (Invitrogen, San Diego, CA, USA) supplemented with 10% FCS at 37°C, and 5% CO_2_. The demethylating agent azacitidine was freshly prepared in ddH_2_O and filter-sterilized.

DNA was extracted from sporadic RCC as described previously [19]. All participants gave informed written consent for research studies and the study was conducted according to the principles expressed in the Declaration of Helsinki and was approved by the relevant Institutional Review Board/Ethics committees. RNA was extracted for 15 patients where sufficient matched RCC tumour and normal tissue was available. Total RNA was isolated from both using RNA-Bee reagent following the manufacturer’s instructions (AMS Biotechnology, Oxford, UK), followed by purification using RNeasy Mini-columns (Qiagen, Crawley, UK).

### *In vitro* assessment of RCC cell line growth

The effect of azacitidine treatment on RCC cell line growth *in vitro* was assessed by colony formation and soft agar assays.

#### ***Colony formation assay***

Cell lines were plated in 75-cm^2^ flasks in DMEM supplemented with 10% FCS at differing densities, depending upon their replication factor, to ensure that both control and azacitidine-treated lines reached approximately 75% confluency at the point of cell counting. Twenty-four hours later, cells were treated with 0.3 μM, 1 μM and 3 μM azacitidine. Subsequent treatments of azacitidine were performed every 24 hours until cells had undergone 72 hours of treatment. After 72 hours treatment with 0.3 μM, 1 μM and 3 μM azacitidine compound, cells were trypsinised and counted. Serial dilutions of 1/500, 1/1,000 and 1/,000 were made from an initial stock of 1×10 [6] treated cells. Cells were seeded into 100-mm tissue-culture dishes and maintained in DMEM and 10% fetal bovine serum. Surviving colonies were stained with 0.4% crystal violet (Sigma) in 50% methanol, 14 to 21 days after initial seeding, and counted. Cells not used in the colony formation assay were snap-frozen in liquid nitrogen for subsequent DNA (Roche, Mannheim, Germany) and RNA (Geneflow, Lichfield, United Kingdom) extraction.

#### ***Soft agar assay***

After treatment with 3 μM azacitidine (as for colony formation assay) cells were seeded into 2 ml DMEM in 10% FCS and 3% agar. Cells were maintained by addition of 200 μl of DMEM in 10% FCS weekly. After 6 weeks of growth, a final count of colonies (>100 μm) was performed.

### Copy number, methylation and expression analyses

#### ***Copy number analysis***

Experiments were performed according to standard protocols for Affymetrix Human SNP Assay 6.0 arrays (Affymetrix Santa Clara, CA, USA). Genomic DNA samples from sporadic primary clear-cell RCC tumours were studied. Genotype and copy number analyses were performed using Affymetrix Genotyping Console version 4.0 with the default settings and the HapMap270 reference model file supplied by Affymetrix. The quality control (QC) call rates ranged from 84.9 to 97.5%. Probe set-level log2 ratios relative to the median of the 270 hapmap reference samples were exported from Genotyping Console. Data within copy number variation regions (Affymetrix Genome-Wide Human SNP Array 6.0 Annotations release 29, July 2009) and on X and Y chromosomes were removed. Autosomal log2 ratios were centred to a median of zero and segmented using GLAD (Hupe *et al*., 2004) with the HaarSeg algorithm [40]. As described previously [41], GISTIC analysis [42] was performed to identify regions of significant copy number gain and loss using the GenePattern public server [43] with the default settings of amplifications threshold of 0.1, deletions threshold of 0.1, join segment size of 4 and qv threshold of 0.25. SNP, gene, and cytogenetic band locations were based on the hg18 (March 2006) genome build (http://genome.ucsc.edu).

#### ***Cell line methylation profiling***

The cell line DNA from all 15 RCC cell lines was assayed using the Illumina Goldengate Methylation Cancer Panel BeadChips (Illumina, San Diego, Ca, USA) as described previously (for a set of primary RCC) [38]. Within this publication three methylated genes had been selected and assessed by direct bisulphite sequencing in several cell lines. The direct bisulphite sequencing demonstrated a high degree of correlation with the GoldenGate array (McNemar test, *P* = 1.0).

#### ***Tumour methylation profiling***

The tumour DNA from the cohort of thirty-eight sporadic renal cell carcinoma patients (a single tumour sample was run twice as a test for reproducibility) and the normal kidney DNA from nine non-cancerous kidneys had been assayed using the Illumina Infinium HumanMethylation27 BeadChips (Illumina). Initial analysis of these data and its confirmation has been previously published [19].

#### ***Cell line expression profiling***

Eleven RCC cell lines (SKRC18, SKRC39, SKRC45, SKRC47, SKRC54, RCC4, 786-O, KTCL26, UMRC2, UMRC3 and CAKI-1) had been treated with or without 5-aza-2′-deoxycytidine treatment for five days and the resulting sets of mRNA had been assayed using the Affymetrix HG-U133plus2 Genechip arrays (Affymetrix) as reported previously [2].

### mRNA expression analysis of selected candidate genes

One microgram of total kidney tumour or kidney cell line RNA was converted to cDNA using Superscript III (Invitrogen) and random hexamer primers (Fermentas UK, York, UK). RT-PCR primers were designed for each gene, such that the primers were always positioned in different exons and had a 56°C annealing temperature. RT-PCR was performed using a touchdown PCR program with five cycles lowered 1°C per cycle down from 61°C to 57°C followed by a further 35 cycles with a final annealing temperature of 56°C. Primer details are available on request. Expression of mRNA was graded as either lost (no band was observed), downregulated (obvious loss) or expressed (no obvious loss).

### The cancer genome atlas (TCGA - http://cancergenome.nih.gov/) data

Data were retrieved from the Cancer Genome Atlas using the TCGA data portal to download the clinical data and Infinium Methylation27 data for the 199 tumour and associated tumour normal samples for which the Infinium Methylation27 arrays had been performed in the TCGA KIRC project. This comprised the following records: TCGA-A3-3306 → 3362 (n = 34), TCGA-B0-3306 → 3362 (n = 6), TCGA-B0-5075 → 5088 (n = 6), TCGA-B2-3923 → 4102 (n = 5), TCGA-B8-4143 → 4154 (n = 2), TCGA-BP-4162 → 4167 (n = 5), TCGA-BP-4158 → 4777 (n = 50), TCGA-BP-4781 → 4807 (n = 11), TCGA-BP-4959 → 5009 (n = 39), TCGA-CJ-4634 → 4644 (n = 9), TCGA-CJ-4635 → 4895 (n = 24), TCGA-CJ-4899 → 4900 (n = 2) and TCGA-CZ-4854 → 4862 (n = 6). Once downloaded, a methylation difference value for each probe in each tumour was calculated as the tumour β-value minus the associated normal β-value. These values were used to assess methylation with a value of +0.3 or greater being considered positive for aberrant hypermethylation.

## Abbreviations

AEBP: Adipocyte enhancer binding protein; CMML: Chronic myelomonocytic leukaemia; DMEM: Dulbecco's modified Eagle's medium; FCS: Fetal calf serum; GAPDH: Glyceraldehyde-3-phosphate dehydrogenase; GISTIC: Genetic identification of significant targets in cancer; HIF: Hypoxia-inducible transcription factor; KIRC: Kidney renal clear cell carcinoma; MAP: Mitogen-activated protein; NIH: National Institutes of Health; PCR: Polymerase chain reaction; QC: Quality control; RCC: Renal cell carcinoma; RGS: Regulator of G-protein signalling; SNP: Single nucleotide polymorphism; TCGA: Cancer Genome Atlas; TSG: Tumour suppressor gene; VHL: Von Hippel-Lindau.

## Competing interests

The authors declare no conflict of interests. Celgene provided financial support for the investigation of demethylating therapy in cell lines but took no part in the analysis or interpretation of the results.

## Authors’ contributions

CJR, MRM, DG and SS carried out the molecular genetic studies. CJR, WW and ERM undertook bioinformatic and statistical analysis. CJR and ERM drafted the manuscript. MB and NC provided reagents and clinical information. CJR, MRM, PN, FL and ERM conceived the study and participated in its design and coordination. All authors read and approved the final manuscript.

## Supplementary Material

Additional file 1: Table S1 Previously published common deletions and amplifications/duplications associated with renal cell carcinoma (RCC). Click here for file

Additional file 2: Figure S1. A-D Schematics of the deleted chromosomes 1, 3p, 14q, 6 and 9. The methylation levels for the selected genes in the 38 sporadic renal cell carcinomas (RCCs) are represented by green ovals for no significant methylation or yellow (β-values ≥0.33) and red (β-values ≥0.4) ovals for positive levels of hypermethylation. The methylation levels for the nine normal kidneys are represented by green ovals for no methylation (β-values <0.25). The degree of re-expression after 5′-aza treatment in the 11 RCC cell lines is represented by green ovals for no significant increase/change or yellow ovals for a positive increase (4-fold or greater) and red ovals for a highly positive increase (8-fold or greater). Other genes of interest were mapped to the chromosome, with black arrows representing known hypermethylated RCC-associated genes and blue arrows representing genes known to be mutated in RCC. The blue star indicates the gene is both mutated and hypermethylated. Click here for file

Additional file 3: Figure S2 Schematics of the Amplified/Duplicated Chromosomes 7, 5q and 20. The methylation levels for the selected genes in the 38 sporadic renal cell carcinomas (RCCs) are represented by green ovals for no significant methylation or yellow (β-values ≥0.33) and red (β-values ≥0.4) ovals for positive levels of hypermethylation. The methylation levels for the nine normal kidneys are represented by green ovals for no methylation (β-values <0.25). The degree of re-expression after 5′-aza treatment in the 11 RCC cell lines is represented by green ovals for no significant increase/change or yellow ovals for a positive increase (4-fold or greater) and red ovals for a highly positive increase (8-fold or greater). Other genes of interest were mapped to the chromosome, with red arrows representing oncogenes, black arrows representing known hypermethylated RCC-associated genes and blue arrows representing genes known to be mutated in RCC. Click here for file

Additional file 4: Figure S3 Assessment of selected methylated Infinium Methylation27 probes in the Cancer Genome Atlas (TCGA) tumour and associated normal pairs. These in-scale diagrams map the position of the Infinium Methylation27 microarray probes for two of the five selected genes, *AEBP1* and *TMEM74*, in relation to their CpG island and first exon. The probes selected for by this analysis are coloured red. For each probe there is a graph of the average β-values for both the tumour and the associated normal with the 199 tumour/associated normal TCGA samples split into those designated methylated or unmethylated. Methylated samples were defined as having an increase in β-value of 0.3 or greater within the tumour compared to the associated normal. The number and percentage of methylated and unmethylated samples are shown, with those demonstrating significant tumour-specific methylation coloured in red. Click here for file

Additional file 5: Table S2 Methylation and clinical data for the 199 Cancer Genome Atlas (TCGA) tumour and associated normal samples. Click here for file

Additional file 6: Figure S4 Additional Kaplan-Meier survival curves for the Cancer Genome Atlas (TCGA) samples dependent on the methylation of the different selected gene probes. These Kaplan-Meier survival curves demonstrate the difference in survival between methylated and unmethylated tumours for individual gene probes for *AEBP1* and *TMEM74***(A)** and for methylation of either four or more or two or more of the five selected gene probes **(B)**. All Kaplan-Meier survival curves were calculated using MedCalc software (http://www.medcalc.org/). Click here for file
