# Supplementary material for: Methylation profiling and evaluation of demethylating therapy in renal cell carcinoma
Source: Clin Epigenetics. 2013 Sep 13;5(1):16. doi: 10.1186/1868-7083-5-16 (PMC3848591; doi:10.1186/1868-7083-5-16)
Supplement: Additional file 1: Table S1 — Previously published common deletions and amplifications/duplications associated with renal cell carcinoma (RCC). [file 1868-7083-5-16-S1.pdf]

# Additional file 1

| Chromosomal Abnormality | | Beroud et al. 1996 | Gunawan et al. 2001 | Yoshimoto et al. 2007 | Toma et al. 2008 | Klatte et al. 2009 | Zhang et al. 2010 | Total |
|---|---|---|---|---|---|---|---|---|
| | **Sample No.** | 118 | 118 | 26 | 22 | 246 | 42 | **572** |
| | **-3p** | 67% | 98% | 81% | 91% | 60% | 90% | **74%** |
| | **+5q** | n/a | 57% | 58% | 45% | 33% | 47% | **43%** |
| | **-14q** | 29% | 63% | 35% | 36% | 28% | 30% | **36%** |
| | **+7** | n/a | 19% | 35% | 32% | 26% | 42% | **26%** |
| | **-8p** | n/a | 34% | n/a | n/a | 20% | 25% | **24%** |
| | **-6q** | n/a | 24% | 31% | 27% | 17% | 17% | **20%** |
| | **-9p** | n/a | 24% | 19% | 32% | 19% | 18% | **19%** |
| | **-4p** | n/a | 14% | n/a | n/a | 15% | 5% | **13%** |

**Additional file 1 Table S1:** Previously Published Common Deletions and Amplifications/ Duplications Associated with Renal Cell Carcinoma (RCC).
