# Supplementary figures and images for: Methylation profiling and evaluation of demethylating therapy in renal cell carcinoma
Source: Clin Epigenetics. 2013 Sep 13;5(1):16. doi: 10.1186/1868-7083-5-16 (PMC3848591; doi:10.1186/1868-7083-5-16)

Additional file 2 Figure S1A

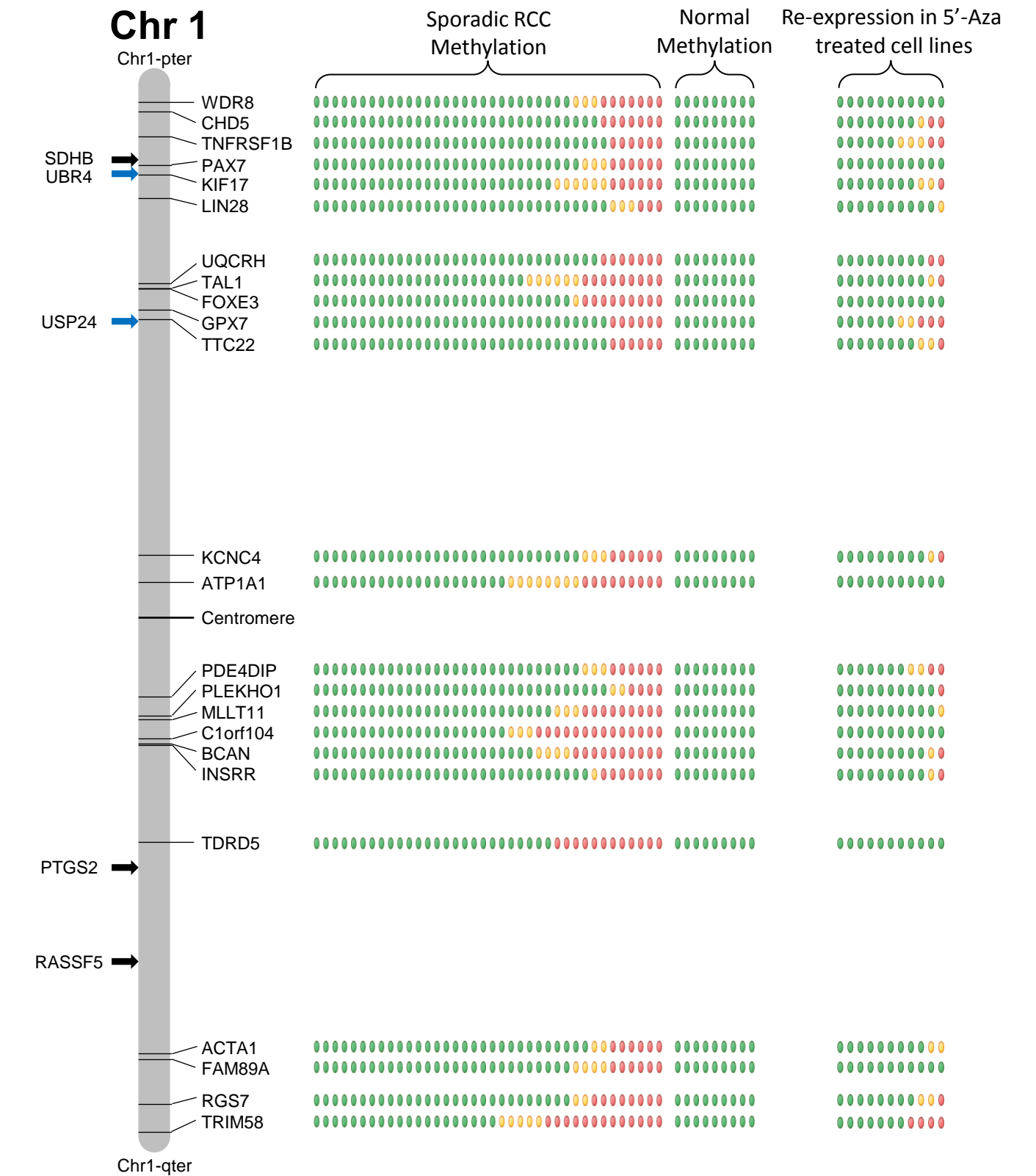

Chr 3p

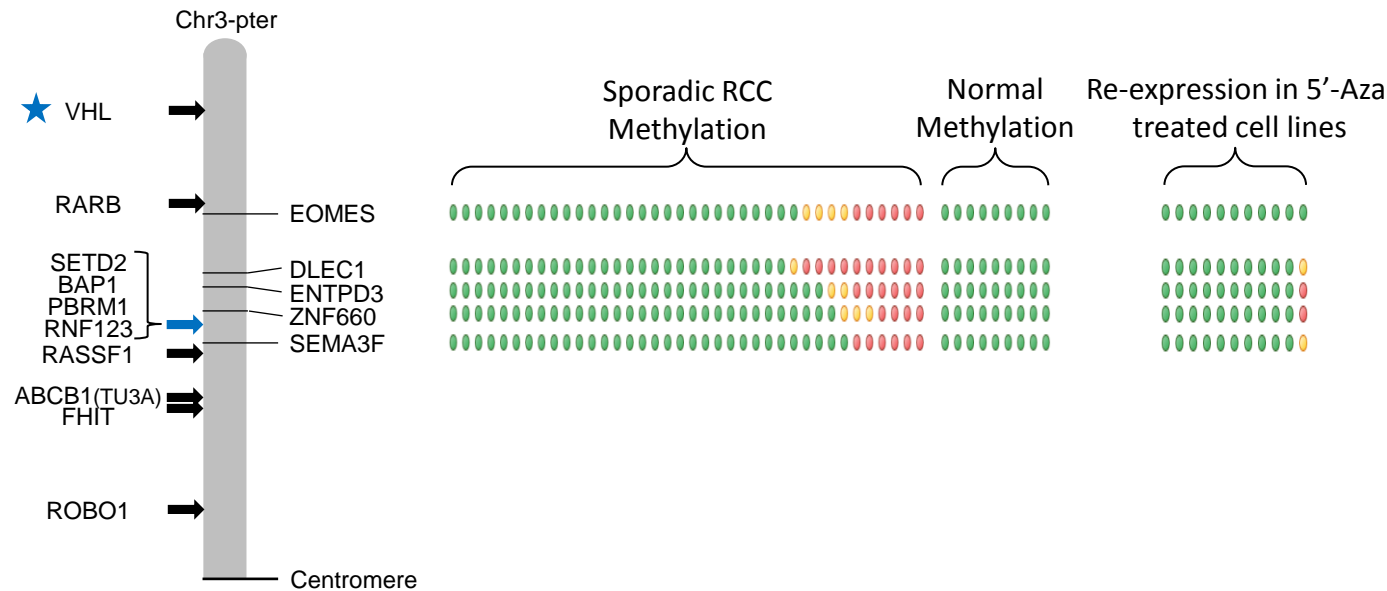

Chr 14q

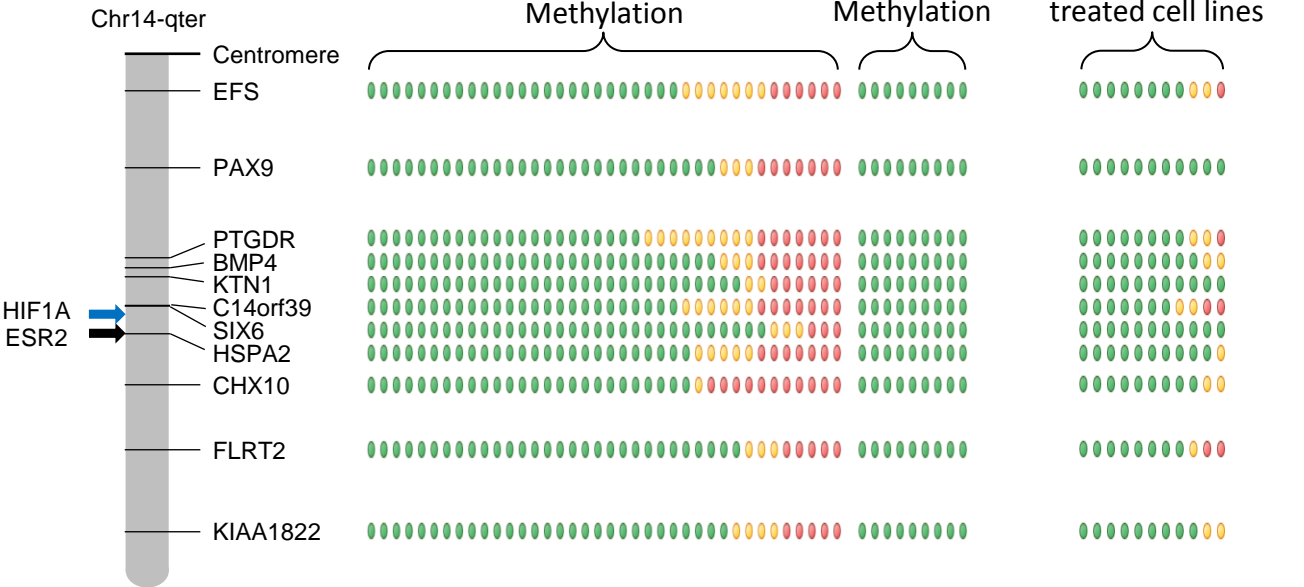

Additional file 2 Figure S1C

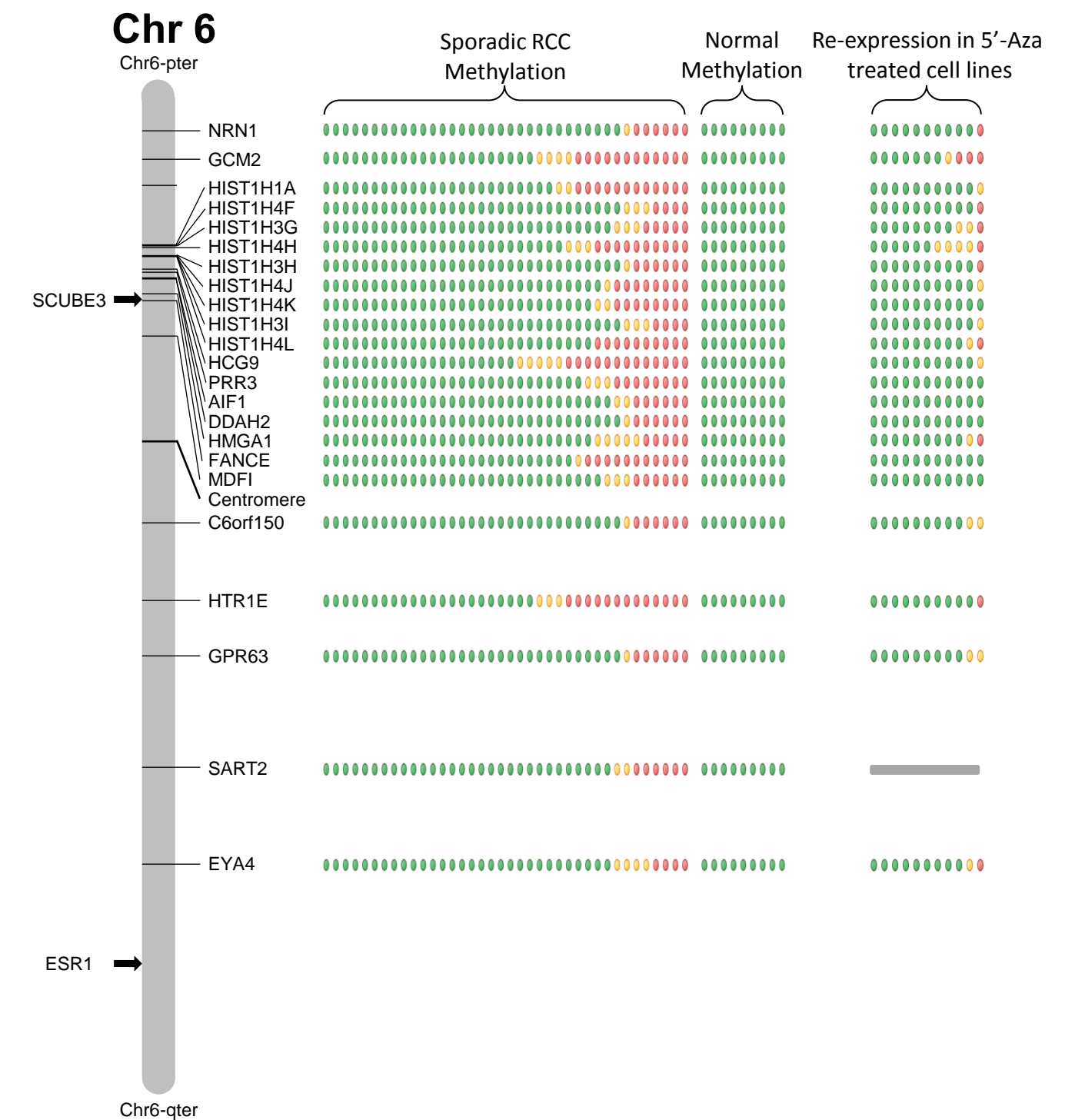

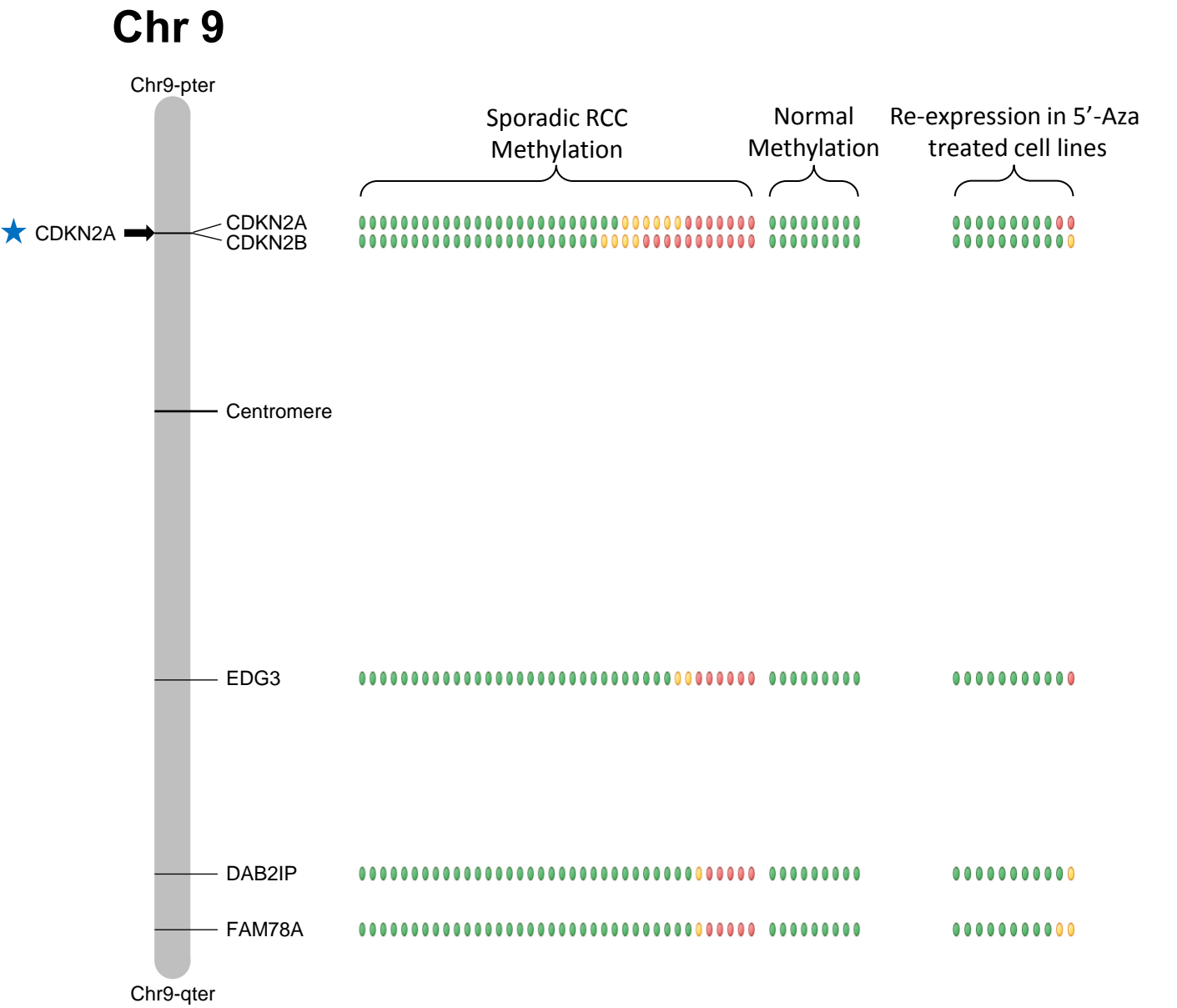

Supplement: Additional file 2: Figure S1. A-D — Schematics of the deleted chromosomes 1, 3p, 14q, 6 and 9. The methylation levels for the selected genes in the 38 sporadic renal cell carcinomas (RCCs) are represented by green ovals for no significant methylation or yellow (β-values ≥0.33) and red (β-values ≥0.4) ovals for positive levels of hypermethylation. The methylation levels for the nine normal kidneys are represented by green ovals for no methylation (β-values <0.25). The degree of re-expression after 5′-aza treatment in the 11 RCC cell lines is represented by green ovals for no significant increase/change or yellow ovals for a positive increase (4-fold or greater) and red ovals for a highly positive increase (8-fold or greater). Other genes of interest were mapped to the chromosome, with black arrows representing known hypermethylated RCC-associated genes and blue arrows representing genes known to be mutated in RCC. The blue star indicates the gene is both mutated and hypermethylated. [file 1868-7083-5-16-S2.pdf]

Chr 5q

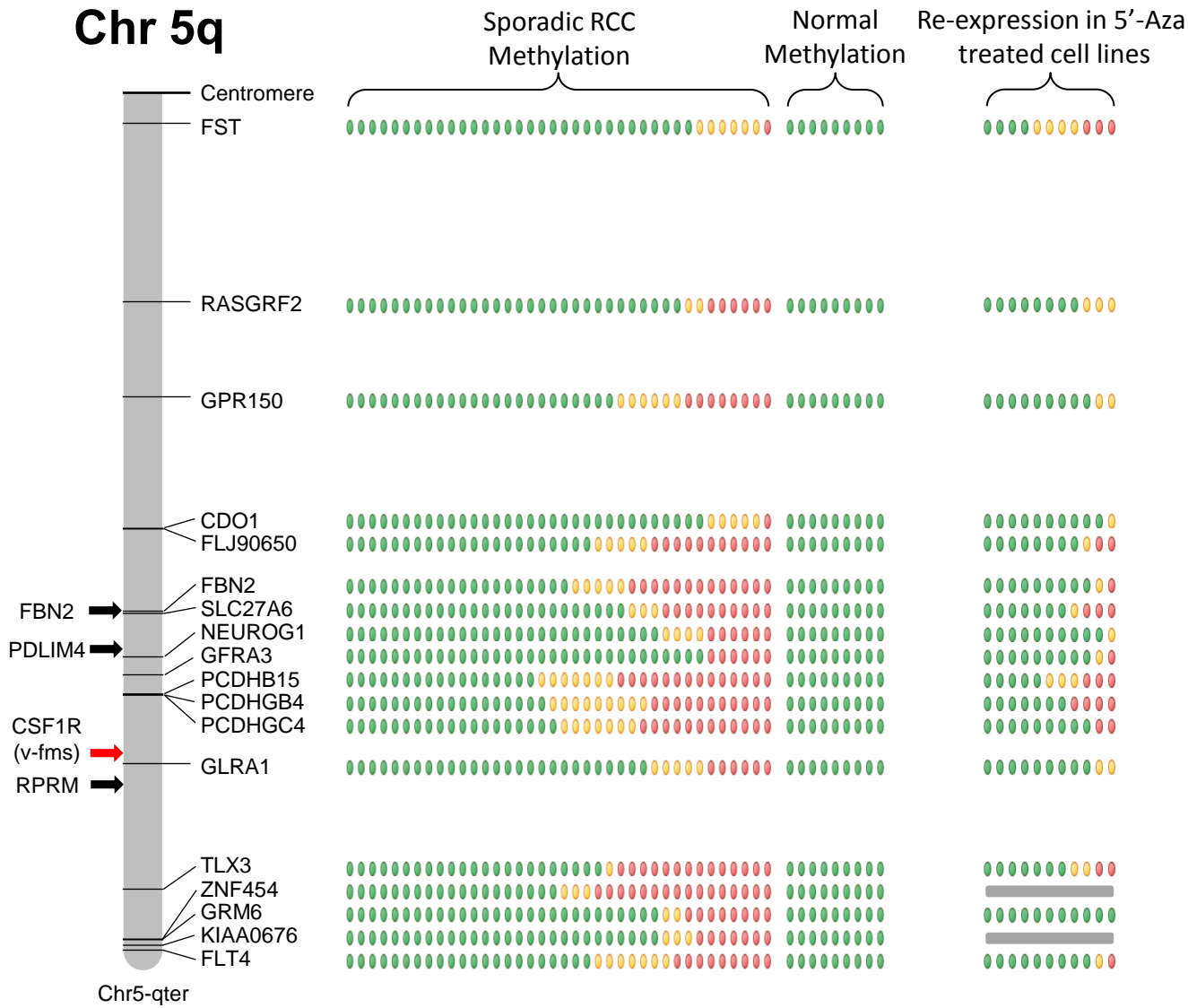

Chr 20

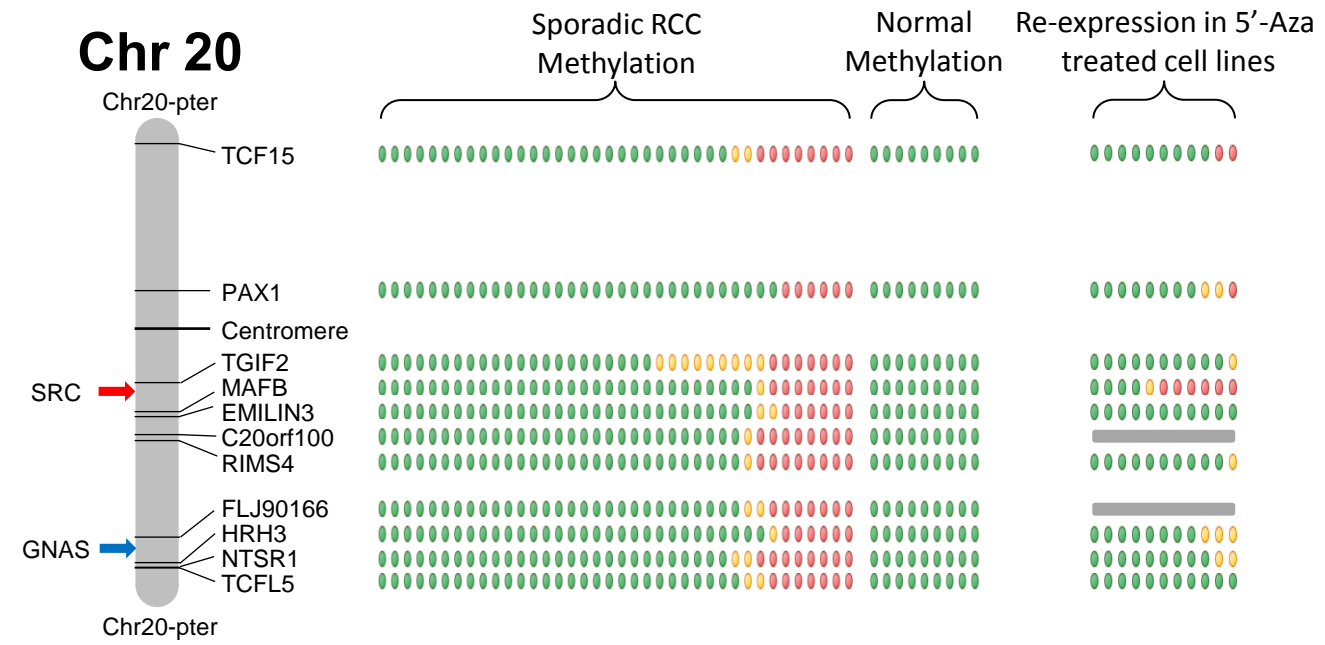

Additional file 3 Figure S2B

Chr 7

Chr7-pter

Chr7-qter

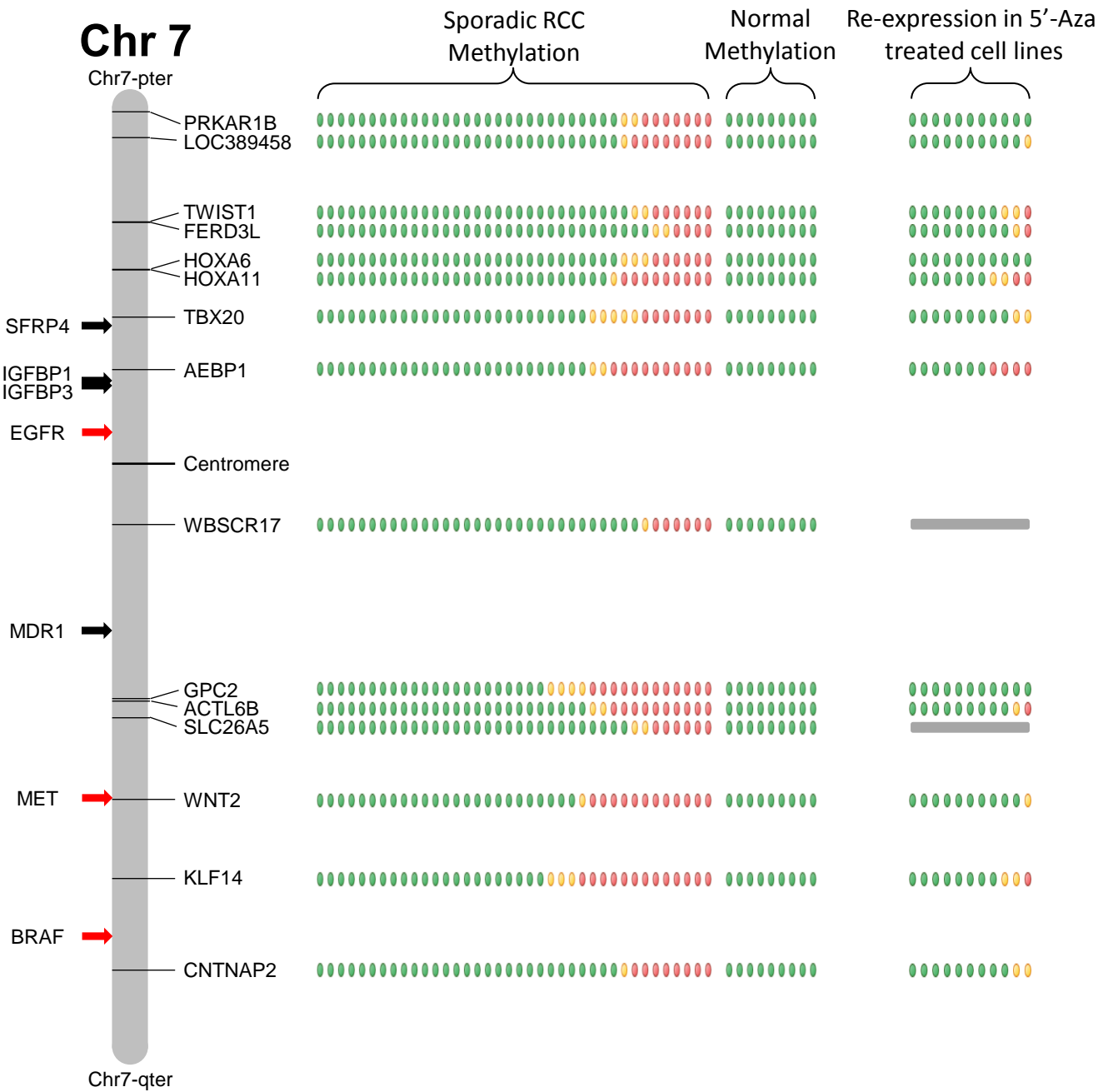

Supplement: Additional file 3: Figure S2 — Schematics of the Amplified/Duplicated Chromosomes 7, 5q and 20. The methylation levels for the selected genes in the 38 sporadic renal cell carcinomas (RCCs) are represented by green ovals for no significant methylation or yellow (β-values ≥0.33) and red (β-values ≥0.4) ovals for positive levels of hypermethylation. The methylation levels for the nine normal kidneys are represented by green ovals for no methylation (β-values <0.25). The degree of re-expression after 5′-aza treatment in the 11 RCC cell lines is represented by green ovals for no significant increase/change or yellow ovals for a positive increase (4-fold or greater) and red ovals for a highly positive increase (8-fold or greater). Other genes of interest were mapped to the chromosome, with red arrows representing oncogenes, black arrows representing known hypermethylated RCC-associated genes and blue arrows representing genes known to be mutated in RCC. [file 1868-7083-5-16-S3.pdf]

Additional file 4 Figure S3

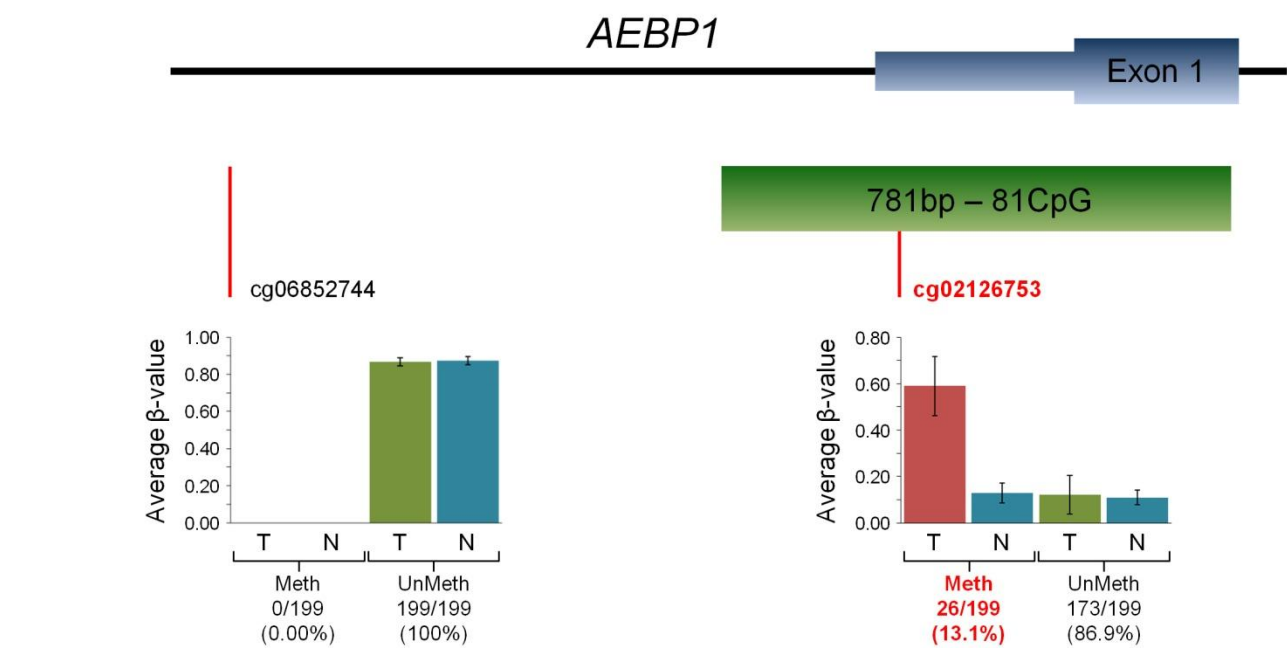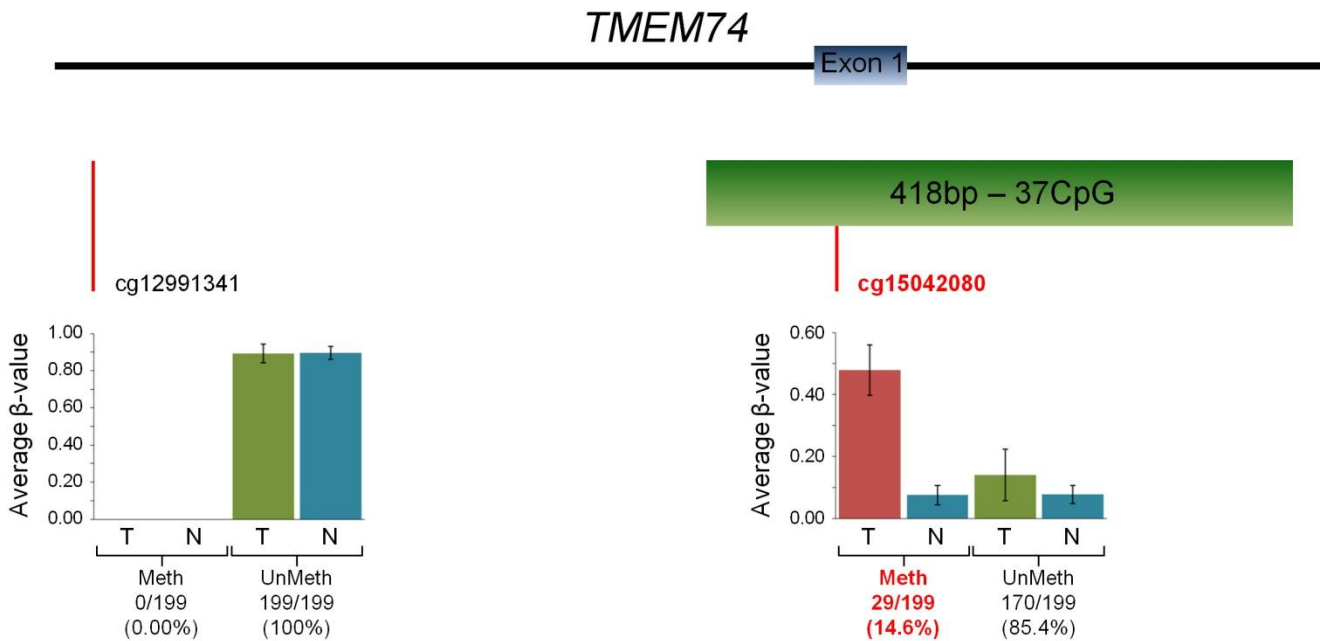

Supplement: Additional file 4: Figure S3 — Assessment of selected methylated Infinium Methylation27 probes in the Cancer Genome Atlas (TCGA) tumour and associated normal pairs. These in-scale diagrams map the position of the Infinium Methylation27 microarray probes for two of the five selected genes, AEBP1 and TMEM74, in relation to their CpG island and first exon. The probes selected for by this analysis are coloured red. For each probe there is a graph of the average β-values for both the tumour and the associated normal with the 199 tumour/associated normal TCGA samples split into those designated methylated or unmethylated. Methylated samples were defined as having an increase in β-value of 0.3 or greater within the tumour compared to the associated normal. The number and percentage of methylated and unmethylated samples are shown, with those demonstrating significant tumour-specific methylation coloured in red. [file 1868-7083-5-16-S4.pdf]

Additional file 6 Figure S4

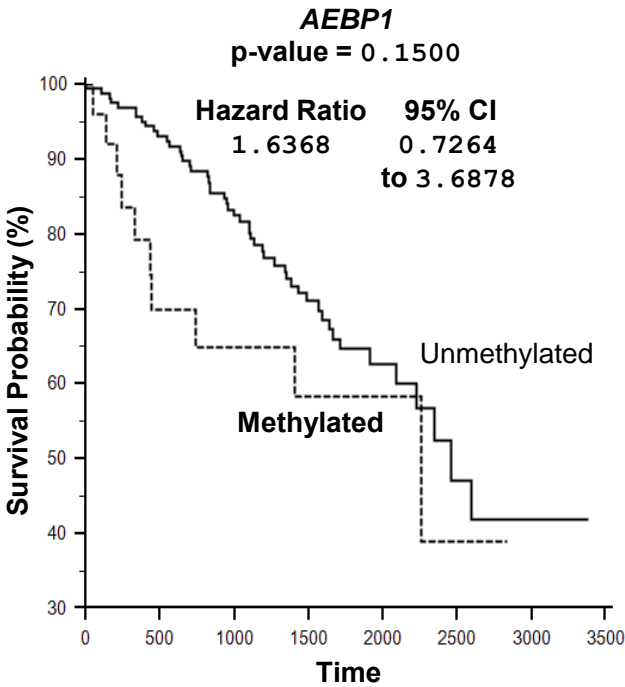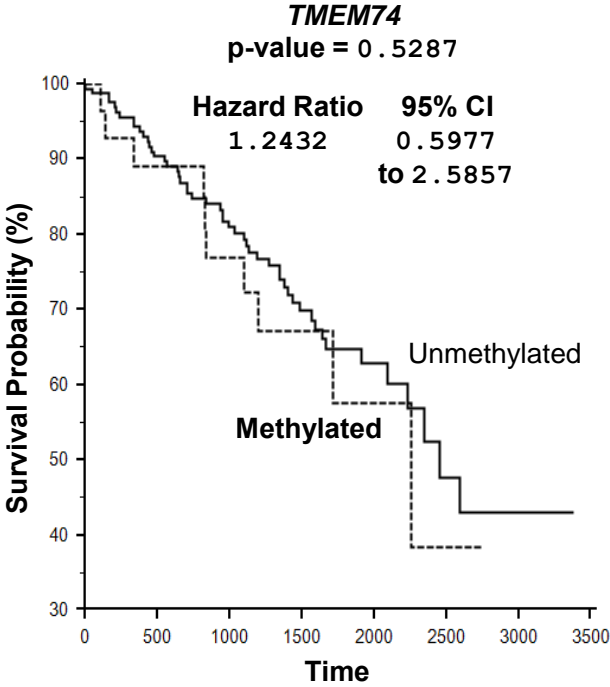

Supplement: Additional file 6: Figure S4 — Additional Kaplan-Meier survival curves for the Cancer Genome Atlas (TCGA) samples dependent on the methylation of the different selected gene probes. These Kaplan-Meier survival curves demonstrate the difference in survival between methylated and unmethylated tumours for individual gene probes for AEBP1 and TMEM74(A) and for methylation of either four or more or two or more of the five selected gene probes (B). All Kaplan-Meier survival curves were calculated using MedCalc software (http://www.medcalc.org/). [file 1868-7083-5-16-S6.pdf]
