# Supplementary material for: Methylation profiling and evaluation of demethylating therapy in renal cell carcinoma
Source: Clin Epigenetics. 2013 Sep 13;5(1):16. doi: 10.1186/1868-7083-5-16 (PMC3848591; doi:10.1186/1868-7083-5-16)
Supplement: Additional file 5: Table S2 — Methylation and clinical data for the 199 Cancer Genome Atlas (TCGA) tumour and associated normal samples. [file 1868-7083-5-16-S5.pdf]

	bcr patient barcode	RGS7 cg21303386	NEF3 cg23290344	TMEM74 cg12991340	GCM2 cg02844545	AEBP1 cg01216753	RGS7 cg24800810	NEF3 cg18267374	TMEM74 cg12991341	GCM2 cg09829319	AEBP1 cg06852744	bcr patient barcode
1	TCGA-A3-3306-11A-01D-0859-05	1	1	1	0	1	0	1	0	0	0	TCGA-A3-3306
2	TCGA-A3-3308-11A-01D-0859-05	0	1	0	0	1	0	0	0	0	0	TCGA-A3-3308
3	TCGA-A3-3311-11A-01D-0859-05	1	0	0	0	0	0	0	0	0	0	TCGA-A3-3311
4	TCGA-A3-3313-11A-01D-0859-05	0	0	0	0	1	0	0	0	0	0	TCGA-A3-3313
5	TCGA-A3-3316-11A-01D-0859-05	1	1	1	0	1	0	1	0	0	0	TCGA-A3-3316
6	TCGA-A3-3317-11A-01D-0859-05	0	1	0	1	0	0	0	0	0	0	TCGA-A3-3317
7	TCGA-A3-3319-11A-01D-0859-05	0	0	0	0	0	0	0	0	0	0	TCGA-A3-3319
8	TCGA-A3-3320-11A-01D-0859-05	0	0	0	0	0	0	0	0	0	0	TCGA-A3-3320
9	TCGA-A3-3322-11A-01D-0859-05	0	0	0	0	0	0	0	0	0	0	TCGA-A3-3322
10	TCGA-A3-3323-11A-01D-0859-05	1	1	1	0	0	0	0	0	0	0	TCGA-A3-3323
11	TCGA-A3-3324-11A-01D-0859-05	0	0	0	0	0	0	0	0	0	0	TCGA-A3-3324
12	TCGA-A3-3325-11A-01D-0859-05	0	0	0	0	0	0	0	0	0	0	TCGA-A3-3325
13	TCGA-A3-3326-11A-01D-0859-05	0	0	0	0	0	0	0	0	0	0	TCGA-A3-3326
14	TCGA-A3-3328-11A-01D-0859-05	0	0	0	0	0	0	1	0	0	0	TCGA-A3-3328
15	TCGA-A3-3329-11A-01D-0859-05	1	0	0	1	0	0	0	0	0	0	TCGA-A3-3329
16	TCGA-A3-3331-11A-01D-0859-05	1	0	1	0	0	0	0	0	0	0	TCGA-A3-3331
17	TCGA-A3-3335-11A-01D-0859-05	1	1	0	1	0	0	0	0	0	0	TCGA-A3-3335
18	TCGA-A3-3336-11A-01D-0859-05	0	0	0	1	0	1	0	0	0	0	TCGA-A3-3336
19	TCGA-A3-3343-11A-01D-0859-05	0	1	1	0	0	0	0	0	0	0	TCGA-A3-3343
20	TCGA-A3-3346-11A-01D-0859-05	1	1	1	1	1	0	0	0	0	0	TCGA-A3-3346
21	TCGA-A3-3352-11A-01D-0859-05	1	1	0	0	0	1	1	0	0	0	TCGA-A3-3352
22	TCGA-A3-3359-11A-01D-0859-05	0	0	0	0	0	0	0	0	0	0	TCGA-A3-3359
23	TCGA-A3-3363-11A-01D-0859-05	0	0	0	0	1	0	0	0	0	0	TCGA-A3-3363
24	TCGA-A3-3365-11A-01D-0859-05	0	0	0	0	0	0	0	0	0	0	TCGA-A3-3365
25	TCGA-A3-3372-11A-01D-0859-05	1	0	0	0	0	0	0	0	0	0	TCGA-A3-3372
26	TCGA-A3-3374-11A-01D-0859-05	0	0	0	0	0	0	0	0	0	0	TCGA-A3-3374
27	TCGA-A3-3378-11A-01D-0859-05	0	1	0	0	0	0	0	0	0	0	TCGA-A3-3378
28	TCGA-A3-3380-11A-01D-0859-05	0	1	0	0	0	0	0	0	0	0	TCGA-A3-3380
29	TCGA-A3-3382-11A-01D-0859-05	1	1	0	1	0	0	0	0	1	0	TCGA-A3-3382
30	TCGA-A3-3383-11A-01D-0859-05	0	0	0	0	0	0	0	0	0	0	TCGA-A3-3383
31	TCGA-A3-3347-11A-01D-1186-05	1	1	0	1	1	0	0	0	0	0	TCGA-A3-3347
32	TCGA-A3-3349-11A-01D-1186-05	0	0	0	0	0	0	0	0	0	0	TCGA-A3-3349
33	TCGA-A3-3351-11A-01D-1186-05	0	0	0	0	0	0	1	0	0	0	TCGA-A3-3351
34	TCGA-A3-3362-11A-01D-1186-05	0	0	0	0	0	0	0	0	0	0	TCGA-A3-3362
35	TCGA-B2-3923-11A-01D-1186-05	0	0	0	0	0	0	0	0	0	0	TCGA-B2-3923
36	TCGA-B2-3924-11A-01D-1186-05	0	0	0	0	0	0	0	0	0	0	TCGA-B2-3924
37	TCGA-B2-4098-11A-01D-1186-05	0	0	0	0	1	0	0	0	0	0	TCGA-B2-4098
38	TCGA-B2-4099-11A-01D-1186-05	0	1	0	0	0	0	0	0	0	0	TCGA-B2-4099
39	TCGA-B2-4102-11A-01D-1186-05	0	0	0	0	0	0	0	0	0	0	TCGA-B2-4102

40	TCGA-B8-4143-11A-01D-1186-05	1	1	0	1	0	0	1	0	1	0	TCGA-B8-4143
41	TCGA-B8-4154-11A-01D-1186-05	1	0	1	0	0	1	0	0	0	0	TCGA-B8-4154
42	TCGA-BP-4161-11A-01D-1186-05	1	1	1	0	1	0	0	0	0	0	TCGA-BP-4161
43	TCGA-BP-4162-11A-01D-1186-05	0	0	0	0	0	0	0	0	0	0	TCGA-BP-4162
44	TCGA-BP-4163-11A-01D-1186-05	0	0	0	1	1	0	0	0	0	0	TCGA-BP-4163
45	TCGA-BP-4164-11A-01D-1186-05	0	0	0	0	0	0	0	0	0	0	TCGA-BP-4164
46	TCGA-BP-4167-11A-01D-1186-05	1	0	0	0	0	0	0	0	0	0	TCGA-BP-4167
47	TCGA-CJ-4634-11A-01D-1186-05	0	0	0	0	0	0	0	0	0	0	TCGA-CJ-4634
48	TCGA-CJ-4636-11A-01D-1186-05	0	0	1	0	0	0	0	0	0	0	TCGA-CJ-4636
49	TCGA-CJ-4637-11A-01D-1186-05	1	1	0	0	0	0	0	0	0	0	TCGA-CJ-4637
50	TCGA-CJ-4638-11A-01D-1186-05	1	1	0	1	1	1	0	0	0	0	TCGA-CJ-4638
51	TCGA-CJ-4639-11A-01D-1186-05	0	0	0	0	0	0	0	0	1	0	TCGA-CJ-4639
52	TCGA-CJ-4640-11A-01D-1186-05	1	0	0	0	0	0	0	0	0	0	TCGA-CJ-4640
53	TCGA-CJ-4641-11A-01D-1186-05	1	0	0	0	0	0	0	0	0	0	TCGA-CJ-4641
54	TCGA-CJ-4643-11A-01D-1186-05	0	0	0	0	0	0	0	0	0	0	TCGA-CJ-4643
55	TCGA-CJ-4644-11A-01D-1186-05	1	0	1	0	0	0	0	0	0	0	TCGA-CJ-4644
56	TCGA-BP-4158-11A-01D-1284-05	0	1	0	0	0	0	0	0	0	0	TCGA-BP-4158
57	TCGA-BP-4159-11A-01D-1284-05	0	1	0	0	0	0	0	0	0	0	TCGA-BP-4159
58	TCGA-BP-4160-11A-01D-1284-05	0	0	0	0	0	0	0	0	0	0	TCGA-BP-4160
59	TCGA-BP-4165-11A-01D-1284-05	1	0	0	0	0	0	0	0	0	0	TCGA-BP-4165
60	TCGA-BP-4166-11A-01D-1284-05	0	0	0	0	0	0	0	0	0	0	TCGA-BP-4166
61	TCGA-BP-4169-11A-01D-1284-05	1	0	0	0	0	0	0	0	0	0	TCGA-BP-4169
62	TCGA-BP-4170-11A-01D-1284-05	0	0	0	0	0	0	0	0	0	0	TCGA-BP-4170
63	TCGA-BP-4173-11A-01D-1284-05	0	0	0	0	0	0	0	0	0	0	TCGA-BP-4173
64	TCGA-BP-4174-11A-01D-1284-05	0	0	0	0	0	1	0	0	0	0	TCGA-BP-4174
65	TCGA-BP-4176-11A-01D-1284-05	1	1	0	1	0	0	0	0	0	0	TCGA-BP-4176
66	TCGA-BP-4325-11A-01D-1284-05	0	0	0	0	0	0	0	0	0	0	TCGA-BP-4325
67	TCGA-BP-4326-11A-01D-1284-05	1	1	1	1	0	1	0	0	0	0	TCGA-BP-4326
68	TCGA-BP-4327-11A-01D-1284-05	0	0	1	0	0	0	0	0	0	0	TCGA-BP-4327
69	TCGA-BP-4329-11A-01D-1284-05	0	1	1	1	1	0	0	0	1	0	TCGA-BP-4329
70	TCGA-BP-4330-11A-01D-1284-05	1	1	1	0	0	0	0	0	0	0	TCGA-BP-4330
71	TCGA-BP-4331-11A-01D-1284-05	0	0	0	0	0	0	1	0	0	0	TCGA-BP-4331
72	TCGA-BP-4332-11A-01D-1284-05	0	0	0	0	0	0	0	0	0	0	TCGA-BP-4332
73	TCGA-BP-4334-11A-01D-1284-05	0	0	0	0	0	0	1	0	0	0	TCGA-BP-4334
74	TCGA-BP-4335-11A-01D-1284-05	1	1	0	0	0	0	0	0	0	0	TCGA-BP-4335
75	TCGA-BP-4337-11A-01D-1284-05	1	0	0	0	0	0	0	0	0	0	TCGA-BP-4337
76	TCGA-BP-4338-11A-01D-1284-05	1	0	0	1	0	1	0	0	0	0	TCGA-BP-4338
77	TCGA-BP-4340-11A-01D-1284-05	0	0	0	0	0	0	0	0	0	0	TCGA-BP-4340
78	TCGA-BP-4341-11A-01D-1284-05	0	1	0	0	0	0	0	0	0	0	TCGA-BP-4341
79	TCGA-BP-4342-11A-01D-1284-05	1	1	1	1	1	0	1	0	0	0	TCGA-BP-4342
80	TCGA-BP-4343-11A-01D-1284-05	1	1	0	0	0	0	0	0	0	0	TCGA-BP-4343
81	TCGA-BP-4344-11A-01D-1284-05	0	0	0	0	0	0	0	0	0	0	TCGA-BP-4344
82	TCGA-BP-4345-11A-01D-1284-05	0	0	0	0	0	0	0	0	0	0	TCGA-BP-4345
83	TCGA-BP-4346-11A-01D-1284-05	1	0	0	0	0	0	0	0	0	0	TCGA-BP-4346
84	TCGA-BP-4347-11A-01D-1284-05	0	0	0	0	0	0	0	0	0	0	TCGA-BP-4347
85	TCGA-BP-4349-11A-01D-1284-05	0	0	0	0	0	1	0	0	0	0	TCGA-BP-4349
86	TCGA-BP-4351-11A-01D-1284-05	0	0	0	0	0	0	0	0	0	0	TCGA-BP-4351

87	TCGA-BP-4352-11A-01D-1284-05	1	1	0	1	1	0	1	0	1	0	TCGA-BP-4352
88	TCGA-BP-4353-11A-01D-1284-05	0	0	0	0	0	0	0	0	0	0	TCGA-BP-4353
89	TCGA-BP-4354-11A-01D-1284-05	1	1	0	0	0	0	1	0	0	0	TCGA-BP-4354
90	TCGA-BP-4355-11A-01D-1284-05	1	0	0	0	0	0	0	0	0	0	TCGA-BP-4355
91	TCGA-BP-4756-11A-01D-1284-05	0	1	0	0	0	0	0	0	0	0	TCGA-BP-4756
92	TCGA-BP-4758-11A-01D-1284-05	0	0	0	0	0	0	0	0	0	0	TCGA-BP-4758
93	TCGA-BP-4759-11A-01D-1284-05	0	0	0	0	0	0	0	0	0	0	TCGA-BP-4759
94	TCGA-BP-4761-11A-01D-1284-05	1	1	0	1	1	1	0	0	0	0	TCGA-BP-4761
95	TCGA-BP-4762-11A-01D-1284-05	0	0	0	0	0	0	0	0	0	0	TCGA-BP-4762
96	TCGA-BP-4763-11A-01D-1284-05	0	1	0	0	0	0	0	0	0	0	TCGA-BP-4763
97	TCGA-BP-4765-11A-01D-1284-05	0	0	0	0	0	0	0	0	0	0	TCGA-BP-4765
98	TCGA-BP-4766-11A-01D-1284-05	0	0	0	0	0	0	0	0	0	0	TCGA-BP-4766
99	TCGA-BP-4768-11A-01D-1284-05	0	0	1	0	1	0	0	0	0	0	TCGA-BP-4768
100	TCGA-BP-4769-11A-01D-1284-05	0	1	0	0	0	0	1	0	0	0	TCGA-BP-4769
101	TCGA-BP-4771-11A-01D-1287-05	0	0	0	0	0	0	0	0	0	0	TCGA-BP-4771
102	TCGA-BP-4774-11A-01D-1287-05	0	0	0	0	0	0	0	0	0	0	TCGA-BP-4774
103	TCGA-BP-4775-11A-01D-1287-05	0	0	0	0	0	0	0	0	0	0	TCGA-BP-4775
104	TCGA-BP-4776-11A-01D-1287-05	0	0	0	0	0	0	0	0	0	0	TCGA-BP-4776
105	TCGA-BP-4777-11A-01D-1287-05	0	0	0	0	0	0	0	0	0	0	TCGA-BP-4777
106	TCGA-B0-4833-11A-01D-1303-05	0	0	0	0	0	0	0	0	0	0	TCGA-B0-4833
107	TCGA-B0-4834-11A-01D-1303-05	0	0	0	0	0	0	0	0	0	0	TCGA-B0-4834
108	TCGA-B0-4836-11A-01D-1303-05	1	1	1	0	0	0	0	0	0	0	TCGA-B0-4836
109	TCGA-B0-4837-11A-01D-1303-05	0	1	0	0	0	0	0	0	0	0	TCGA-B0-4837
110	TCGA-B0-4838-11A-01D-1303-05	0	0	0	0	0	0	0	0	0	0	TCGA-B0-4838
111	TCGA-B0-4839-11A-01D-1303-05	0	0	0	0	0	0	0	0	0	0	TCGA-B0-4839
112	TCGA-BP-4781-11A-01D-1303-05	0	1	0	0	1	0	0	0	0	0	TCGA-BP-4781
113	TCGA-BP-4784-11A-01D-1303-05	0	0	0	0	0	0	0	0	0	0	TCGA-BP-4784
114	TCGA-BP-4787-11A-01D-1303-05	1	0	0	0	0	0	0	0	0	0	TCGA-BP-4787
115	TCGA-BP-4789-11A-01D-1303-05	0	0	0	0	0	0	0	0	0	0	TCGA-BP-4789
116	TCGA-BP-4790-11A-01D-1303-05	0	0	0	0	0	0	0	0	0	0	TCGA-BP-4790
117	TCGA-BP-4797-11A-01D-1303-05	0	0	0	0	0	0	0	0	0	0	TCGA-BP-4797
118	TCGA-BP-4798-11A-01D-1303-05	0	1	0	1	0	0	0	0	0	0	TCGA-BP-4798
119	TCGA-BP-4799-11A-01D-1303-05	1	1	0	1	0	0	0	0	0	0	TCGA-BP-4799
120	TCGA-BP-4803-11A-01D-1303-05	0	0	0	0	0	0	0	0	0	0	TCGA-BP-4803
121	TCGA-BP-4804-11A-01D-1303-05	0	0	0	0	0	0	0	0	0	0	TCGA-BP-4804
122	TCGA-BP-4807-11A-01D-1303-05	0	0	0	0	0	0	0	0	0	0	TCGA-BP-4807
123	TCGA-CJ-4635-11B-01D-1303-05	1	0	0	0	1	0	0	0	0	0	TCGA-CJ-4635
124	TCGA-CJ-4642-11A-01D-1303-05	1	0	0	0	0	0	0	0	0	0	TCGA-CJ-4642
125	TCGA-CJ-4868-11A-01D-1303-05	1	1	0	1	0	0	0	0	1	0	TCGA-CJ-4868
126	TCGA-CJ-4870-11A-01D-1303-05	0	0	1	0	0	0	0	0	0	0	TCGA-CJ-4870
127	TCGA-CJ-4871-11A-01D-1303-05	1	0	1	0	0	0	0	0	0	0	TCGA-CJ-4871
128	TCGA-CJ-4872-11A-01D-1303-05	1	1	0	0	0	0	0	0	0	0	TCGA-CJ-4872
129	TCGA-CJ-4873-11A-01D-1303-05	0	0	0	0	0	0	0	0	0	0	TCGA-CJ-4873
130	TCGA-CJ-4874-11A-01D-1303-05	0	0	0	0	0	0	0	0	0	0	TCGA-CJ-4874
131	TCGA-CJ-4875-11A-01D-1303-05	0	0	0	0	0	0	0	0	0	0	TCGA-CJ-4875
132	TCGA-CJ-4876-11A-01D-1303-05	1	1	0	1	0	1	0	0	0	0	TCGA-CJ-4876
133	TCGA-CJ-4878-11A-01D-1303-05	1	0	0	1	0	0	0	0	0	0	TCGA-CJ-4878

134	TCGA-CJ-4881-11A-01D-1303-05	1	0	0	0	1	0	0	0	0	0	TCGA-CJ-4881
135	TCGA-CJ-4884-11A-01D-1303-05	1	0	0	1	0	0	0	0	0	0	TCGA-CJ-4884
136	TCGA-CJ-4885-11A-01D-1303-05	0	0	0	0	0	0	0	0	0	0	TCGA-CJ-4885
137	TCGA-CJ-4886-11A-01D-1303-05	1	0	0	0	0	0	0	0	0	0	TCGA-CJ-4886
138	TCGA-CJ-4887-11A-01D-1303-05	1	0	0	0	0	0	0	0	0	0	TCGA-CJ-4887
139	TCGA-CJ-4888-11A-01D-1303-05	1	1	0	0	0	0	0	0	0	0	TCGA-CJ-4888
140	TCGA-CJ-4889-11A-01D-1303-05	0	0	0	0	0	0	0	0	0	0	TCGA-CJ-4889
141	TCGA-CJ-4890-11A-01D-1303-05	0	0	1	0	0	0	0	0	0	0	TCGA-CJ-4890
142	TCGA-CJ-4891-11A-01D-1303-05	1	1	1	0	0	0	0	0	0	0	TCGA-CJ-4891
143	TCGA-CJ-4892-11A-01D-1303-05	0	0	0	0	0	0	0	0	0	0	TCGA-CJ-4892
144	TCGA-CJ-4893-11A-01D-1303-05	0	0	0	0	0	0	0	0	0	0	TCGA-CJ-4893
145	TCGA-CJ-4894-11A-01D-1303-05	0	0	0	0	0	0	1	0	0	0	TCGA-CJ-4894
146	TCGA-CJ-4895-11A-01D-1303-05	1	1	1	0	0	0	1	0	0	0	TCGA-CJ-4895
147	TCGA-CZ-4854-11A-01D-1303-05	1	1	0	0	1	0	0	0	0	0	TCGA-CZ-4854
148	TCGA-CZ-4857-11A-01D-1303-05	1	1	0	1	0	1	0	0	0	0	TCGA-CZ-4857
149	TCGA-CZ-4858-11A-01D-1303-05	0	0	0	1	0	0	0	0	0	0	TCGA-CZ-4858
150	TCGA-CZ-4860-11A-01D-1303-05	1	1	0	1	1	0	0	0	0	0	TCGA-CZ-4860
151	TCGA-CZ-4861-11A-01D-1303-05	0	1	0	1	1	0	1	0	0	0	TCGA-CZ-4861
152	TCGA-CZ-4862-11A-01D-1303-05	0	0	0	0	0	0	0	0	0	0	TCGA-CZ-4862
153	TCGA-B0-5075-11A-01D-1332-05	1	0	0	0	0	0	0	0	0	0	TCGA-B0-5075
154	TCGA-B0-5077-11A-01D-1332-05	0	0	0	0	0	0	0	0	0	0	TCGA-B0-5077
155	TCGA-B0-5081-11A-01D-1332-05	1	0	0	0	0	0	0	0	0	0	TCGA-B0-5081
156	TCGA-B0-5084-11A-01D-1332-05	0	0	0	0	1	0	0	0	0	0	TCGA-B0-5084
157	TCGA-B0-5085-11A-01D-1332-05	0	0	1	0	0	0	1	0	0	0	TCGA-B0-5085
158	TCGA-B0-5088-11A-01D-1332-05	0	0	0	0	0	0	0	0	0	0	TCGA-B0-5088
159	TCGA-BP-4959-11A-01D-1332-05	0	0	0	0	0	0	0	0	0	0	TCGA-BP-4959
160	TCGA-BP-4960-11A-01D-1332-05	1	1	0	0	0	0	0	0	0	0	TCGA-BP-4960
161	TCGA-BP-4961-11A-01D-1332-05	1	0	0	0	0	0	0	0	0	0	TCGA-BP-4961
162	TCGA-BP-4962-11A-01D-1332-05	1	1	0	0	0	0	0	0	0	0	TCGA-BP-4962
163	TCGA-BP-4963-11A-01D-1332-05	1	0	0	0	0	0	0	0	0	0	TCGA-BP-4963
164	TCGA-BP-4964-11A-01D-1332-05	1	0	0	0	1	0	1	0	0	0	TCGA-BP-4964
165	TCGA-BP-4965-11A-01D-1332-05	0	0	0	0	0	0	0	0	0	0	TCGA-BP-4965
166	TCGA-BP-4967-11A-01D-1332-05	0	0	1	0	0	0	0	0	0	0	TCGA-BP-4967
167	TCGA-BP-4968-11A-01D-1332-05	1	0	0	0	0	0	1	0	0	0	TCGA-BP-4968
168	TCGA-BP-4969-11A-01D-1332-05	0	1	0	0	0	0	0	0	0	0	TCGA-BP-4969
169	TCGA-BP-4970-11A-01D-1332-05	0	0	0	0	0	0	0	0	0	0	TCGA-BP-4970
170	TCGA-BP-4971-11A-01D-1332-05	0	0	0	0	0	0	0	0	0	0	TCGA-BP-4971
171	TCGA-BP-4972-11A-01D-1332-05	0	0	0	0	0	0	0	0	0	0	TCGA-BP-4972
172	TCGA-BP-4973-11A-01D-1332-05	0	0	0	0	0	1	0	0	0	0	TCGA-BP-4973
173	TCGA-BP-4974-11A-01D-1332-05	0	0	0	0	0	0	0	0	0	0	TCGA-BP-4974
174	TCGA-BP-4975-11A-01D-1332-05	0	0	0	0	0	0	0	0	0	0	TCGA-BP-4975
175	TCGA-BP-4976-11A-01D-1332-05	0	1	0	0	0	0	0	0	0	0	TCGA-BP-4976
176	TCGA-BP-4977-11A-01D-1332-05	0	0	0	0	0	0	0	0	0	0	TCGA-BP-4977
177	TCGA-BP-4981-11A-01D-1332-05	0	0	0	0	0	0	0	0	0	0	TCGA-BP-4981
178	TCGA-BP-4982-11A-01D-1332-05	1	0	0	0	0	0	0	0	0	0	TCGA-BP-4982
179	TCGA-BP-4983-11A-01D-1332-05	1	1	0	0	1	0	0	0	0	0	TCGA-BP-4983
180	TCGA-BP-4985-11A-01D-1332-05	1	1	0	1	0	0	0	0	0	0	TCGA-BP-4985

181	TCGA-BP-4986-11A-01D-1332-05	0	0	0	0	0	0	0	0	0	0	TCGA-BP-4986
182	TCGA-BP-4987-11A-01D-1332-05	0	0	0	0	0	0	0	0	0	0	TCGA-BP-4987
183	TCGA-BP-4988-11A-01D-1332-05	0	0	1	0	0	0	0	0	0	0	TCGA-BP-4988
184	TCGA-BP-4989-11A-01D-1332-05	1	1	0	0	0	0	0	0	0	0	TCGA-BP-4989
185	TCGA-BP-4991-11A-01D-1332-05	0	0	1	0	0	0	0	0	0	0	TCGA-BP-4991
186	TCGA-BP-4992-11A-01D-1332-05	0	0	0	0	1	0	0	0	0	0	TCGA-BP-4992
187	TCGA-BP-4994-11A-01D-1332-05	0	0	0	0	0	0	0	0	0	0	TCGA-BP-4994
188	TCGA-BP-4995-11A-01D-1332-05	0	0	1	0	0	0	0	0	0	0	TCGA-BP-4995
189	TCGA-BP-4998-11A-01D-1332-05	0	0	0	0	0	0	0	0	0	0	TCGA-BP-4998
190	TCGA-BP-4999-11A-01D-1332-05	0	0	0	0	0	0	0	0	0	0	TCGA-BP-4999
191	TCGA-BP-5000-11A-01D-1332-05	1	1	0	0	0	0	0	0	0	0	TCGA-BP-5000
192	TCGA-BP-5001-11A-01D-1332-05	0	0	0	0	0	0	0	0	0	0	TCGA-BP-5001
193	TCGA-BP-5004-11A-01D-1332-05	0	1	0	0	0	0	0	0	0	0	TCGA-BP-5004
194	TCGA-BP-5006-11A-01D-1332-05	0	0	0	0	0	0	0	0	0	0	TCGA-BP-5006
195	TCGA-BP-5007-11A-01D-1332-05	0	0	0	0	0	0	0	0	0	0	TCGA-BP-5007
196	TCGA-BP-5008-11A-01D-1332-05	0	0	0	0	0	0	0	0	0	0	TCGA-BP-5008
197	TCGA-BP-5009-11A-01D-1332-05	1	1	1	1	0	1	0	0	0	0	TCGA-BP-5009
198	TCGA-CJ-4899-11A-01D-1332-05	0	0	0	0	0	0	0	0	0	0	TCGA-CJ-4899
199	TCGA-CJ-4900-11A-01D-1332-05	0	1	1	0	0	0	0	0	0	0	TCGA-CJ-4900
		70	60	29	29	26	13	18	0	6	0	
		RGS7	NEF3	TMEM74	GCM2	AEBP1	RGS7	NEF3	TMEM74	GCM2	AEBP1	
		cg21303386	cg23290344	cg12991340	cg02844545	cg01216753	cg24800810	cg18267374	cg12991341	cg09829319	cg06852744	

Additional file 5 Table S2: Methylation and Clinical Data for the 199 TCGA Tumour and Associated Normal Sa

age at initial pathologic diagnosis	days to last known alive or death	gender	histological type
67	1120	MALE	Kidney Clear Cell Renal Carcinoma
77	16	FEMALE	Kidney Clear Cell Renal Carcinoma
59	1190	MALE	Kidney Clear Cell Renal Carcinoma
59	735	MALE	Kidney Clear Cell Renal Carcinoma
57	1493	MALE	Kidney Clear Cell Renal Carcinoma
67	1491	MALE	Kidney Clear Cell Renal Carcinoma
70	1130	MALE	Kidney Clear Cell Renal Carcinoma
52	1508	FEMALE	Kidney Clear Cell Renal Carcinoma
51	1477	MALE	Kidney Clear Cell Renal Carcinoma
53	1105	MALE	Kidney Clear Cell Renal Carcinoma
51	1186	MALE	Kidney Clear Cell Renal Carcinoma
52	751	MALE	Kidney Clear Cell Renal Carcinoma
47	1137	MALE	Kidney Clear Cell Renal Carcinoma
null	null	null	null
75	706	MALE	Kidney Clear Cell Renal Carcinoma
86	1506	FEMALE	Kidney Clear Cell Renal Carcinoma
41	664	MALE	Kidney Clear Cell Renal Carcinoma
75	1043	FEMALE	Kidney Clear Cell Renal Carcinoma
79	944	MALE	Kidney Clear Cell Renal Carcinoma
68	137	MALE	Kidney Clear Cell Renal Carcinoma
74	549	MALE	Kidney Clear Cell Renal Carcinoma
null	null	null	null
50	319	MALE	Kidney Clear Cell Renal Carcinoma
46	872	MALE	Kidney Clear Cell Renal Carcinoma
64	735	MALE	Kidney Clear Cell Renal Carcinoma
51	1313	FEMALE	Kidney Clear Cell Renal Carcinoma
60	630	MALE	Kidney Clear Cell Renal Carcinoma
54	567	MALE	Kidney Clear Cell Renal Carcinoma
69	574	FEMALE	Kidney Clear Cell Renal Carcinoma
52	861	MALE	Kidney Clear Cell Renal Carcinoma
76	1097	FEMALE	Kidney Clear Cell Renal Carcinoma
34	1385	FEMALE	Kidney Clear Cell Renal Carcinoma
42	910	MALE	Kidney Clear Cell Renal Carcinoma
60	1559	FEMALE	Kidney Clear Cell Renal Carcinoma
59	178	MALE	Kidney Clear Cell Renal Carcinoma
73	194	MALE	Kidney Clear Cell Renal Carcinoma
72	50	FEMALE	Kidney Clear Cell Renal Carcinoma
83	110	MALE	Kidney Clear Cell Renal Carcinoma
61	15	MALE	Kidney Clear Cell Renal Carcinoma

66	709	FEMALE	Kidney Clear Cell Renal Carcinoma
73	255	FEMALE	Kidney Clear Cell Renal Carcinoma
74	2746	MALE	Kidney Clear Cell Renal Carcinoma
65	3074	FEMALE	Kidney Clear Cell Renal Carcinoma
60	2830	FEMALE	Kidney Clear Cell Renal Carcinoma
51	992	FEMALE	Kidney Clear Cell Renal Carcinoma
59	2718	MALE	Kidney Clear Cell Renal Carcinoma
60	1820	FEMALE	Kidney Clear Cell Renal Carcinoma
51	1924	MALE	Kidney Clear Cell Renal Carcinoma
52	2227	FEMALE	Kidney Clear Cell Renal Carcinoma
46	431	FEMALE	Kidney Clear Cell Renal Carcinoma
49	2308	FEMALE	Kidney Clear Cell Renal Carcinoma
49	1966	MALE	Kidney Clear Cell Renal Carcinoma
55	1661	FEMALE	Kidney Clear Cell Renal Carcinoma
67	1792	FEMALE	Kidney Clear Cell Renal Carcinoma
48	336	FEMALE	Kidney Clear Cell Renal Carcinoma
69	3377	MALE	Kidney Clear Cell Renal Carcinoma
70	2592	MALE	Kidney Clear Cell Renal Carcinoma
67	2881	MALE	Kidney Clear Cell Renal Carcinoma
64	2946	FEMALE	Kidney Clear Cell Renal Carcinoma
69	13	MALE	Kidney Clear Cell Renal Carcinoma
76	701	FEMALE	Kidney Clear Cell Renal Carcinoma
72	2343	FEMALE	Kidney Clear Cell Renal Carcinoma
47	1893	MALE	Kidney Clear Cell Renal Carcinoma
49	1879	MALE	Kidney Clear Cell Renal Carcinoma
64	1955	MALE	Kidney Clear Cell Renal Carcinoma
64	2964	FEMALE	Kidney Clear Cell Renal Carcinoma
53	1624	FEMALE	Kidney Clear Cell Renal Carcinoma
75	108	FEMALE	Kidney Clear Cell Renal Carcinoma
75	845	MALE	Kidney Clear Cell Renal Carcinoma
60	1864	FEMALE	Kidney Clear Cell Renal Carcinoma
52	2453	MALE	Kidney Clear Cell Renal Carcinoma
36	1133	MALE	Kidney Clear Cell Renal Carcinoma
56	645	MALE	Kidney Clear Cell Renal Carcinoma
65	460	FEMALE	Kidney Clear Cell Renal Carcinoma
76	2	FEMALE	Kidney Clear Cell Renal Carcinoma
43	2746	MALE	Kidney Clear Cell Renal Carcinoma
70	562	FEMALE	Kidney Clear Cell Renal Carcinoma
67	1589	MALE	Kidney Clear Cell Renal Carcinoma
79	2256	MALE	Kidney Clear Cell Renal Carcinoma
64	1912	MALE	Kidney Clear Cell Renal Carcinoma
75	1666	FEMALE	Kidney Clear Cell Renal Carcinoma
62	1409	MALE	Kidney Clear Cell Renal Carcinoma
57	1485	MALE	Kidney Clear Cell Renal Carcinoma
74	1367	MALE	Kidney Clear Cell Renal Carcinoma
68	364	FEMALE	Kidney Clear Cell Renal Carcinoma
51	961	FEMALE	Kidney Clear Cell Renal Carcinoma

74	332	FEMALE	Kidney Clear Cell Renal Carcinoma
61	375	MALE	Kidney Clear Cell Renal Carcinoma
40	1034	MALE	Kidney Clear Cell Renal Carcinoma
59	953	FEMALE	Kidney Clear Cell Renal Carcinoma
62	370	FEMALE	Kidney Clear Cell Renal Carcinoma
40	2208	MALE	Kidney Clear Cell Renal Carcinoma
50	2324	MALE	Kidney Clear Cell Renal Carcinoma
57	182	MALE	Kidney Clear Cell Renal Carcinoma
42	1343	MALE	Kidney Clear Cell Renal Carcinoma
79	1270	FEMALE	Kidney Clear Cell Renal Carcinoma
43	2184	MALE	Kidney Clear Cell Renal Carcinoma
43	1467	FEMALE	Kidney Clear Cell Renal Carcinoma
72	400	FEMALE	Kidney Clear Cell Renal Carcinoma
63	1876	MALE	Kidney Clear Cell Renal Carcinoma
62	161	MALE	Kidney Clear Cell Renal Carcinoma
57	1884	FEMALE	Kidney Clear Cell Renal Carcinoma
55	1842	FEMALE	Kidney Clear Cell Renal Carcinoma
52	410	MALE	Kidney Clear Cell Renal Carcinoma
46	1731	MALE	Kidney Clear Cell Renal Carcinoma
null	null	null	null
49	2090	MALE	Kidney Clear Cell Renal Carcinoma
null	null	null	null
63	1378	MALE	Kidney Clear Cell Renal Carcinoma
69	834	FEMALE	Kidney Clear Cell Renal Carcinoma
80	1638	FEMALE	Kidney Clear Cell Renal Carcinoma
78	2080	MALE	Kidney Clear Cell Renal Carcinoma
67	1854	FEMALE	Kidney Clear Cell Renal Carcinoma
59	479	FEMALE	Kidney Clear Cell Renal Carcinoma
48	1489	MALE	Kidney Clear Cell Renal Carcinoma
76	1111	MALE	Kidney Clear Cell Renal Carcinoma
34	1107	MALE	Kidney Clear Cell Renal Carcinoma
74	334	MALE	Kidney Clear Cell Renal Carcinoma
70	1133	MALE	Kidney Clear Cell Renal Carcinoma
42	168	MALE	Kidney Clear Cell Renal Carcinoma
57	1128	MALE	Kidney Clear Cell Renal Carcinoma
42	211	MALE	Kidney Clear Cell Renal Carcinoma
48	1416	MALE	Kidney Clear Cell Renal Carcinoma
47	1617	MALE	Kidney Clear Cell Renal Carcinoma
42	645	MALE	Kidney Clear Cell Renal Carcinoma
58	1497	FEMALE	Kidney Clear Cell Renal Carcinoma
63	2422	MALE	Kidney Clear Cell Renal Carcinoma
51	1435	MALE	Kidney Clear Cell Renal Carcinoma
85	2258	FEMALE	Kidney Clear Cell Renal Carcinoma
73	2283	FEMALE	Kidney Clear Cell Renal Carcinoma
67	2353	MALE	Kidney Clear Cell Renal Carcinoma
null	null	null	null
71	2186	FEMALE	Kidney Clear Cell Renal Carcinoma

41	2013	MALE	Kidney Clear Cell Renal Carcinoma
72	993	FEMALE	Kidney Clear Cell Renal Carcinoma
64	2125	MALE	Kidney Clear Cell Renal Carcinoma
42	1951	FEMALE	Kidney Clear Cell Renal Carcinoma
48	931	MALE	Kidney Clear Cell Renal Carcinoma
59	1566	MALE	Kidney Clear Cell Renal Carcinoma
63	1945	FEMALE	Kidney Clear Cell Renal Carcinoma
72	2085	MALE	Kidney Clear Cell Renal Carcinoma
57	818	FEMALE	Kidney Clear Cell Renal Carcinoma
65	1520	FEMALE	Kidney Clear Cell Renal Carcinoma
76	749	FEMALE	Kidney Clear Cell Renal Carcinoma
null	null	MALE	Kidney Clear Cell Renal Carcinoma
62	1200	MALE	Kidney Clear Cell Renal Carcinoma
68	1404	MALE	Kidney Clear Cell Renal Carcinoma
56	1432	MALE	Kidney Clear Cell Renal Carcinoma
39	1943	MALE	Kidney Clear Cell Renal Carcinoma
60	205	MALE	Kidney Clear Cell Renal Carcinoma
63	445	MALE	Kidney Clear Cell Renal Carcinoma
46	1843	MALE	Kidney Clear Cell Renal Carcinoma
77	637	FEMALE	Kidney Clear Cell Renal Carcinoma
77	1345	MALE	Kidney Clear Cell Renal Carcinoma
79	402	FEMALE	Kidney Clear Cell Renal Carcinoma
33	239	MALE	Kidney Clear Cell Renal Carcinoma
75	838	FEMALE	Kidney Clear Cell Renal Carcinoma
53	654	MALE	Kidney Clear Cell Renal Carcinoma
49	2660	MALE	Kidney Clear Cell Renal Carcinoma
46	2172	MALE	Kidney Clear Cell Renal Carcinoma
47	1935	MALE	Kidney Clear Cell Renal Carcinoma
58	1785	MALE	Kidney Clear Cell Renal Carcinoma
63	1834	MALE	Kidney Clear Cell Renal Carcinoma
54	1862	FEMALE	Kidney Clear Cell Renal Carcinoma
46	1871	MALE	Kidney Clear Cell Renal Carcinoma
76	204	MALE	Kidney Clear Cell Renal Carcinoma
40	1745	MALE	Kidney Clear Cell Renal Carcinoma
63	1794	FEMALE	Kidney Clear Cell Renal Carcinoma
44	432	MALE	Kidney Clear Cell Renal Carcinoma
40	1476	MALE	Kidney Clear Cell Renal Carcinoma
43	1501	FEMALE	Kidney Clear Cell Renal Carcinoma
47	1384	MALE	Kidney Clear Cell Renal Carcinoma
58	215	MALE	Kidney Clear Cell Renal Carcinoma
40	1433	MALE	Kidney Clear Cell Renal Carcinoma
77	1632	MALE	Kidney Clear Cell Renal Carcinoma
57	454	MALE	Kidney Clear Cell Renal Carcinoma
75	1097	FEMALE	Kidney Clear Cell Renal Carcinoma
42	1013	MALE	Kidney Clear Cell Renal Carcinoma
67	1412	FEMALE	Kidney Clear Cell Renal Carcinoma
72	951	MALE	Kidney Clear Cell Renal Carcinoma

75	785	MALE	Kidney Clear Cell Renal Carcinoma
41	1124	FEMALE	Kidney Clear Cell Renal Carcinoma
72	827	MALE	Kidney Clear Cell Renal Carcinoma
58	117	MALE	Kidney Clear Cell Renal Carcinoma
54	1413	MALE	Kidney Clear Cell Renal Carcinoma
66	500	MALE	Kidney Clear Cell Renal Carcinoma
54	1307	MALE	Kidney Clear Cell Renal Carcinoma
68	1371	MALE	Kidney Clear Cell Renal Carcinoma
49	931	MALE	Kidney Clear Cell Renal Carcinoma
56	1266	MALE	Kidney Clear Cell Renal Carcinoma
40	563	MALE	Kidney Clear Cell Renal Carcinoma
43	1177	FEMALE	Kidney Clear Cell Renal Carcinoma
53	1126	MALE	Kidney Clear Cell Renal Carcinoma
61	840	MALE	Kidney Clear Cell Renal Carcinoma
45	1140	MALE	Kidney Clear Cell Renal Carcinoma
46	1071	MALE	Kidney Clear Cell Renal Carcinoma
52	1102	MALE	Kidney Clear Cell Renal Carcinoma
42	1527	MALE	Kidney Clear Cell Renal Carcinoma
69	1713	FEMALE	Kidney Clear Cell Renal Carcinoma

l Samples.

lymphnode pathologic spread	number of lymphnodes examined	number of lymphnodes positive	person neoplasm cancer status	primary tumor pathologic spread	primary tumor pathologic spread for analysis
N0	null	null	null	T1b	T1
N0	null	null	TUMOR FREE	T3b	T3
NX	null	null	TUMOR FREE	T1	T1
N0	null	null	TUMOR FREE	T1b	T1
NX	null	null	TUMOR FREE	T2	T2
N0	null	null	TUMOR FREE	T2	T2
NX	null	null	TUMOR FREE	T1b	T1
NX	null	null	TUMOR FREE	T1b	T1
NX	null	null	TUMOR FREE	T1a	T1
NX	null	null	TUMOR FREE	T1b	T1
NX	null	null	TUMOR FREE	T1b	T1
NX	null	null	TUMOR FREE	T1a	T1
NX	null	null	TUMOR FREE	T1a	T1
null	null	null	null	null	null
N0	null	null	TUMOR FREE	T1b	T1
N0	null	null	TUMOR FREE	T1	T1
N0	null	null	TUMOR FREE	T2a	T2
NX	null	null	WITH TUMOR	T1	T1
N0	1	0	TUMOR FREE	T2	T2
NX	null	null	WITH TUMOR	T1b	T1
N0	null	null	null	T3a	T3
null	null	null	null	null	null
N0	null	null	TUMOR FREE	T2	T2
NX	null	null	null	T1a	T1
NX	null	null	TUMOR FREE	T3	T3
N0	null	null	null	T1b	T1
N0	null	null	TUMOR FREE	T1	T1
N0	null	null	TUMOR FREE	T1	T1
NX	null	null	WITH TUMOR	T1b	T1
NX	null	null	null	T1	T1
N1	1	1	null	T1b	T1
N0	null	null	TUMOR FREE	T1b	T1
N0	null	null	TUMOR FREE	T2a	T2
N0	null	null	TUMOR FREE	T1a	T1
NX	null	null	TUMOR FREE	T2	T2
NX	null	null	TUMOR FREE	T1b	T1
NX	null	null	TUMOR FREE	T1b	T1
NX	null	null	TUMOR FREE	T1a	T1
NX	null	null	TUMOR FREE	T1b	T1

N0	2	0	WITH TUMOR	null	
NX	null	null	TUMOR FREE	T1a	T1
NX	null	null	WITH TUMOR	T1b	T1
N0	3	0	TUMOR FREE	T1b	T1
N0	9	0	TUMOR FREE	T3a	T3
NX	null	null	TUMOR FREE	T3a	T3
NX	null	null	TUMOR FREE	T3a	T3
NX	0	0	TUMOR FREE	T1b	T1
N0	4	0	TUMOR FREE	T3a	T3
NX	null	null	WITH TUMOR	T2b	T2
N1	3	2	WITH TUMOR	T3a	T3
N0	null	null	TUMOR FREE	T2	T2
N0	null	null	TUMOR FREE	T3a	T3
NX	0	0	WITH TUMOR	T3a	T3
N0	null	null	TUMOR FREE	T2b	T2
N0	null	null	WITH TUMOR	T3a	T3
N0	5	0	TUMOR FREE	T1b	T1
N0	null	0	WITH TUMOR	T1b	T1
N0	3	0	TUMOR FREE	T3a	T3
N0	2	0	WITH TUMOR	T1b	T1
N0	10	0	TUMOR FREE	T3a	T3
N0	2	0	WITH TUMOR	T2	T2
N0	13	0	TUMOR FREE	T1b	T1
N0	5	0	TUMOR FREE	T2	T2
N0	3	0	TUMOR FREE	T2	T2
NX	null	null	TUMOR FREE	T1b	T1
N0	null	null	TUMOR FREE	T1b	T1
N0	3	0	TUMOR FREE	T1b	T1
N0	6	0	TUMOR FREE	T2	T2
N0	1	0	TUMOR FREE	T3a	T3
N0	13	0	TUMOR FREE	T3a	T3
N0	null	null	WITH TUMOR	T1a	T1
N0	null	null	TUMOR FREE	T3a	T3
N0	1	0	TUMOR FREE	T3a	T3
N0	null	null	WITH TUMOR	T3a	T3
N0	1	0	TUMOR FREE	T3b	T3
N0	null	null	TUMOR FREE	T1b	T1
N0	null	null	TUMOR FREE	T1b	T1
NX	null	null	WITH TUMOR	T3a	T3
N0	7	0	WITH TUMOR	T2	T2
N0	7	0	WITH TUMOR	T3a	T3
NX	null	null	TUMOR FREE	T1a	T1
N0	1	0	TUMOR FREE	T3b	T3
N0	21	0	TUMOR FREE	T3b	T3
NX	null	null	TUMOR FREE	T3b	T3
NX	null	null	null	T1a	T1
N0	17	0	WITH TUMOR	T3a	T3

N0	10	0	WITH TUMOR	T3b	T3
N0	null	null	TUMOR FREE	T1	T1
N1	21	1	WITH TUMOR	T4	T4
NX	null	null	null	T3a	T3
N0	1	0	TUMOR FREE	T1b	T1
NX	null	null	TUMOR FREE	T1a	T1
NX	null	null	TUMOR FREE	T1a	T1
N1	16	2	TUMOR FREE	T3a	T3
NX	null	null	TUMOR FREE	T1a	T1
NX	null	null	TUMOR FREE	T1a	T1
NX	null	null	TUMOR FREE	T1a	T1
NX	null	null	TUMOR FREE	T1a	T1
N0	7	0	TUMOR FREE	T1a	T1
NX	null	null	TUMOR FREE	T1a	T1
N0	5	0	TUMOR FREE	T3a	T3
NX	null	null	TUMOR FREE	T1a	T1
NX	null	null	WITH TUMOR	T1a	T1
NX	null	null	TUMOR FREE	T1a	T1
NX	null	null	TUMOR FREE	T1a	T1
null	null	null	null	null	null
N0	0	0	TUMOR FREE	T1a	T1
null	null	null	null	null	null
N0	0	0	WITH TUMOR	T1b	T1
N0	0	0	TUMOR FREE	T1	T1
N0	0	0	TUMOR FREE	T1b	T1
NX	null	null	TUMOR FREE	T1a	T1
NX	null	null	TUMOR FREE	T1a	T1
N0	19	0	WITH TUMOR	T3a	T3
NX	null	null	TUMOR FREE	T1a	T1
NX	null	null	TUMOR FREE	T1a	T1
N0	5	0	TUMOR FREE	T3b	T3
N0	30	0	WITH TUMOR	T3b	T3
N0	2	0	WITH TUMOR	T3b	T3
N0	3	0	WITH TUMOR	T3b	T3
NX	null	null	TUMOR FREE	T1a	T1
NX	null	null	TUMOR FREE	T1a	T1
NX	null	null	TUMOR FREE	T1b	T1
NX	null	null	TUMOR FREE	T2	T2
N0	14	0	WITH TUMOR	T3a	T3
NX	0	null	TUMOR FREE	T3a	T3
NX	0	null	TUMOR FREE	T3a	T3
N0	1	0	TUMOR FREE	T1b	T1
N0	3	0	TUMOR FREE	T3a	T3
N0	0	null	TUMOR FREE	T1b	T1
NX	0	null	WITH TUMOR	T3a	T3
null	null	null	null	null	null
NX	0	null	TUMOR FREE	T3a	T3

NX	0	null	WITH TUMOR	T3a	T3
NX	0	null	TUMOR FREE	T3a	T3
NX	0	null	WITH TUMOR	T3a	T3
NX	0	null	TUMOR FREE	T1a	T1
NX	0	null	WITH TUMOR	T3a	T3
NX	0	null	WITH TUMOR	T3a	T3
NX	1	0	TUMOR FREE	T1a	T1
N0	1	0	WITH TUMOR	T3a	T3
N0	5	0	TUMOR FREE	T3c	T3
N0	2	0	TUMOR FREE	T1b	T1
NX	null	null	TUMOR FREE	T1b	T1
N0	3	0	WITH TUMOR	T3a	T3
NX	null	null	WITH TUMOR	T3a	T3
N0	2	0	TUMOR FREE	T1b	T1
N0	3	0	WITH TUMOR	T3a	T3
NX	0	null	WITH TUMOR	T2	T2
NX	0	null	WITH TUMOR	T4	T4
NX	0	null	WITH TUMOR	T2	T2
NX	0	null	TUMOR FREE	T1b	T1
N0	0	0	TUMOR FREE	T3a	T3
N0	0	0	TUMOR FREE	T1a	T1
N0	0	0	WITH TUMOR	T3b	T3
N1	1	1	WITH TUMOR	T3a	T3
N0	0	0	TUMOR FREE	T3a	T3
N0	0	0	TUMOR FREE	T1b	T1
NX	null	null	TUMOR FREE	T1b	T1
N0	9	0	TUMOR FREE	T2	T2
NX	null	null	TUMOR FREE	T1a	T1
NX	null	null	TUMOR FREE	T2	T2
NX	null	null	TUMOR FREE	T1b	T1
N0	11	0	TUMOR FREE	T1a	T1
NX	null	null	TUMOR FREE	T1a	T1
N0	5	0	TUMOR FREE	T3a	T3
N0	3	0	TUMOR FREE	T1b	T1
NX	null	null	TUMOR FREE	T1a	T1
N0	2	0	TUMOR FREE	T1a	T1
N0	5	0	TUMOR FREE	T3a	T3
NX	null	null	TUMOR FREE	T3a	T3
NX	null	null	TUMOR FREE	T3a	T3
N0	7	0	WITH TUMOR	T2	T2
NX	null	null	TUMOR FREE	T1b	T1
NX	null	null	TUMOR FREE	T1a	T1
NX	null	null	TUMOR FREE	T1b	T1
NX	null	null	TUMOR FREE	T3a	T3
NX	null	null	TUMOR FREE	T1b	T1
NX	null	null	TUMOR FREE	T3a	T3
N0	22	0	WITH TUMOR	T3a	T3

N0	4	0	TUMOR FREE	T1a	T1
NX	null	null	TUMOR FREE	T1b	T1
N0	1	0	TUMOR FREE	T1a	T1
N0	10	0	TUMOR FREE	T3a	T3
NX	null	null	TUMOR FREE	T1a	T1
NX	null	null	TUMOR FREE	T1b	T1
NX	null	null	TUMOR FREE	T1a	T1
N0	5	0	TUMOR FREE	T1b	T1
NX	null	null	TUMOR FREE	T1a	T1
NX	null	null	TUMOR FREE	T1a	T1
NX	null	null	TUMOR FREE	T1b	T1
NX	null	null	TUMOR FREE	T1b	T1
NX	null	null	TUMOR FREE	T1a	T1
N0	2	0	TUMOR FREE	T1a	T1
N0	10	0	TUMOR FREE	T2	T2
NX	null	null	TUMOR FREE	T1a	T1
NX	null	null	WITH TUMOR	T1b	T1
NX	null	null	TUMOR FREE	T1b	T1
N1	14	1	WITH TUMOR	T4	T4

tumor grade	vital status	white cell count result	year of initial pathologic diagnosis
G3	LIVING	null	2005
G2	LIVING	Normal	2006
G2	DECEASED	null	2005
G3	DECEASED	null	2005
G3	LIVING	null	2005
G2	LIVING	null	2005
G2	LIVING	null	2006
G1	LIVING	null	2005
G2	LIVING	null	2006
G1	LIVING	null	2006
G3	LIVING	null	2006
G2	LIVING	null	2006
G1	LIVING	null	2006
null	null	null	null
G2	LIVING	null	2007
G2	LIVING	null	2005
G4	LIVING	null	2006
G4	LIVING	null	2007
G3	LIVING	null	2005
G3	DECEASED	null	2005
G3	DECEASED	null	2005
null	null	null	null
G2	LIVING	null	2006
G2	LIVING	null	2006
G2	LIVING	null	2007
G2	LIVING	null	2005
G3	LIVING	null	2006
G2	LIVING	null	2007
G3	LIVING	null	2007
G2	LIVING	null	2006
G2	LIVING	null	2005
G2	LIVING	null	2006
G2	LIVING	null	2007
G2	LIVING	null	2005
G2	LIVING	Normal	2010
G2	LIVING	Normal	2010
G2	DECEASED	Normal	2010
G2	LIVING	Normal	2010
G2	LIVING	Normal	2010

G3	DECEASED	Normal	2002
G2	LIVING	Low	2010
null	LIVING	Elevated	2002
G2	LIVING	Elevated	2002
G3	LIVING	Normal	2002
G2	DECEASED	Normal	2002
G2	LIVING	Elevated	2003
G2	LIVING	Normal	2004
G3	LIVING	Normal	2004
G4	DECEASED	Normal	2004
G4	DECEASED	Normal	2004
G2	LIVING	Normal	2004
G4	LIVING	Normal	2004
G4	DECEASED	Normal	2005
G3	LIVING	Normal	2005
G3	DECEASED	Normal	2004
G2	LIVING	Normal	2001
G2	DECEASED	Elevated	2002
G2	LIVING	Normal	2002
G1	LIVING	Elevated	2002
G3	LIVING	Elevated	2003
G2	DECEASED	Elevated	2003
G2	DECEASED	Elevated	2003
G3	LIVING	Normal	2004
G3	LIVING	Elevated	2004
G2	LIVING	Elevated	2005
G2	LIVING	Elevated	2001
G2	LIVING	Elevated	2002
G2	DECEASED	Normal	2002
G2	LIVING	Normal	2002
G2	LIVING	Elevated	2002
G2	DECEASED	Normal	2002
G2	LIVING	Elevated	2002
G3	LIVING	Elevated	2003
G3	DECEASED	Elevated	2003
G4	DECEASED	Normal	2003
G3	LIVING	Elevated	2003
G2	DECEASED	Normal	2003
G2	DECEASED	Elevated	2003
G3	DECEASED	Normal	2003
G3	DECEASED	Elevated	2003
G2	LIVING	Elevated	2006
G3	LIVING	Normal	2006
G3	DECEASED	Elevated	2006
G2	LIVING	Elevated	2006
G2	LIVING	Normal	2006
G2	LIVING	Normal	2006

G4	DECEASED	Elevated	2007
G2	DECEASED	Normal	2007
G4	DECEASED	Normal	2006
G4	DECEASED	Elevated	2008
G2	LIVING	Elevated	2003
G2	LIVING	Elevated	2004
G2	LIVING	Elevated	2004
G4	LIVING	Elevated	2004
G3	DECEASED	Elevated	2004
G2	DECEASED	Elevated	2004
G2	LIVING	Elevated	2004
G3	LIVING	Elevated	2004
G2	LIVING	Normal	2005
G2	LIVING	Normal	2005
G4	DECEASED	Normal	2005
G2	LIVING	Normal	2005
G2	LIVING	Elevated	2005
G2	LIVING	Elevated	2005
G3	LIVING	Elevated	2005
null	null	null	null
G3	DECEASED	Normal	2003
null	null	null	null
G3	DECEASED	Elevated	2003
G3	DECEASED	Normal	2003
GX	DECEASED	Normal	2003
G3	LIVING	Normal	2005
G2	LIVING	Normal	2005
G4	DECEASED	Normal	2007
G2	LIVING	Normal	2007
G2	DECEASED	Normal	2007
G3	LIVING	Normal	2007
G4	DECEASED	Normal	2007
G3	DECEASED	Elevated	2007
G4	DECEASED	Normal	2007
G2	LIVING	Normal	2007
G3	LIVING	Normal	2008
G3	LIVING	Normal	2004
G2	LIVING	Normal	2005
G3	DECEASED	null	2005
G2	LIVING	Normal	2004
G4	LIVING	Normal	2004
G4	LIVING	Normal	2004
G3	LIVING	Normal	2004
G3	LIVING	Normal	2004
G3	LIVING	Normal	2004
null	null	null	null
G2	LIVING	Normal	2004

G3	LIVING	Normal	2004
null	LIVING	Normal	2005
G3	LIVING	Normal	2005
G3	LIVING	Low	2005
G3	DECEASED	Normal	2005
G4	DECEASED	Normal	2005
G4	LIVING	Normal	2005
G4	LIVING	Normal	2005
G4	DECEASED	Normal	2005
G2	LIVING	Normal	2005
G3	LIVING	Normal	2005
G2	DECEASED	Normal	null
G4	DECEASED	Normal	2005
G2	DECEASED	Normal	2005
G3	DECEASED	Normal	2005
G4	LIVING	Normal	2005
G4	DECEASED	Normal	2005
G2	DECEASED	Low	2005
G2	LIVING	Elevated	2006
G2	DECEASED	Normal	2005
G3	DECEASED	Normal	2005
G2	DECEASED	Elevated	2005
G3	DECEASED	Normal	2006
G3	DECEASED	Normal	2004
G2	DECEASED	Normal	2006
G3	LIVING	Elevated	2003
G3	LIVING	Normal	2004
G2	LIVING	Elevated	2005
G2	LIVING	Elevated	2005
G3	LIVING	Elevated	2005
G2	LIVING	Elevated	2005
G2	LIVING	Normal	2005
G2	LIVING	Elevated	2005
G3	LIVING	Elevated	2006
G2	LIVING	Elevated	2006
G3	LIVING	Normal	2006
G3	LIVING	Normal	2006
G3	LIVING	Elevated	2006
G3	LIVING	Elevated	2006
G4	DECEASED	Elevated	2006
G3	LIVING	Elevated	2006
G3	LIVING	Normal	2006
G3	LIVING	Elevated	2006
G3	DECEASED	Elevated	2006
G3	LIVING	Elevated	2006
G4	LIVING	Elevated	2006
G4	DECEASED	Elevated	2006

G3	LIVING	Elevated	2006
G2	LIVING	Elevated	2006
G2	DECEASED	Elevated	2007
G3	LIVING	Elevated	2007
G2	LIVING	Elevated	2007
G4	LIVING	Normal	2007
G3	LIVING	Elevated	2007
G3	LIVING	Elevated	2007
G3	LIVING	Normal	2007
G2	LIVING	Elevated	2007
G3	LIVING	Elevated	2007
G2	LIVING	Elevated	2007
G3	LIVING	Elevated	2007
G2	LIVING	Elevated	2007
G2	LIVING	Elevated	2007
G2	LIVING	Elevated	2007
G3	DECEASED	Elevated	2007
G2	LIVING	Normal	2006
G4	DECEASED	Elevated	2006
